# Crystal structure of the inclusion complex 25-benzo­ylmeth­oxy-5,11,17,23-tetra-*tert*-butyl-26,27,28-trihy­droxy-2,8,14,20-tetra­thia­calix[4]arene–tetra­ethyl­ammonium chloride (1/1)

**DOI:** 10.1107/S2056989015018617

**Published:** 2015-10-10

**Authors:** Mehmet Akkurt, Jerry P. Jasinski, Shaaban K. Mohamed, Omran A. Omran, Mustafa R. Albayati

**Affiliations:** a Department of Physics, Faculty of Sciences, Erciyes University, 38039 Kayseri, Turkey; b Department of Chemistry, Keene State College, 229 Main Street, Keene, NH 03435-2001, USA; c Chemistry and Environmental Division, Manchester Metropolitan University, Manchester M1 5GD, England; d Chemistry Department, Faculty of Science, Minia University, 61519 El-Minia, Egypt; e Medical Laboratory Department, College of Science, Majmaah University, 11932, Saudi Arabia; f Chemistry Department, Faculty of Science, Sohag University, 82524 Sohag, Egypt; g Kirkuk University, College of Science, Department of Chemistry, Kirkuk, Iraq

**Keywords:** crystal structure, co-crystal, *p*-*tert*-butyl­thia­calix[4]arene, phase transfer catalysis, tetra­ethyl­ammonium chloride, alkyl­ation, hydrogen bonding

## Abstract

The asymmetric unit of the title compound, C_48_H_54_O_5_S_4_·N(C_2_H_5_)_4_^+^·Cl^−^, contains two tetra-*tert*-butyl-[(benzo­yl)meth­oxy]-trihy­droxy-tetra­thia­calix[4]arene mol­ecules, two tetra­ethyl­ammonium cations and two chloride anions. The two calixarene molecules in the asymmetric unit each display a cone conformation. There are no significant differences between the two independent molecules. The guest species do not sit within the calixarene ‘buckets’. In the crystal, extensive O—H⋯O, O—H⋯S and O—H⋯Cl hydrogen bonds and weak C—H⋯O, C—H⋯S and C—H⋯Cl inter­actions link the thia­calixarene mol­ecules, tetra­ethyl­ammonium cations and chloride anions, forming a three-dimensional network encompassing channels running parallel to the *a*-axis direction. The structure contains a solvent-accessible void of 76 (3) Å^3^, but no solvent mol­ecule could reasonably be located. The crystal studied was an inversion twin with a 0.57 (8):0.43 (8) domain ratio.

## Related literature   

For chemistry background and applications of thia­calixarenes, see: Gutsche (1998 ▸); Shokova & Kovalev (2003 ▸); Stoikov *et al.* (2003 ▸); Agrawal & Pancholi (2007 ▸); Ben Ali *et al.* (2001 ▸); Desroches *et al.* (2003 ▸); Higuchi *et al.* (2000 ▸); Iki & Miyano (2001 ▸); Morohashi *et al.* (2002 ▸); Odo *et al.* (2000 ▸, 2001 ▸); Omran & Anti­pin (2014 ▸). Many calixarene derivatives are being used in both environmental and biomedical monitoring, see: McMahon *et al.* (2003 ▸).
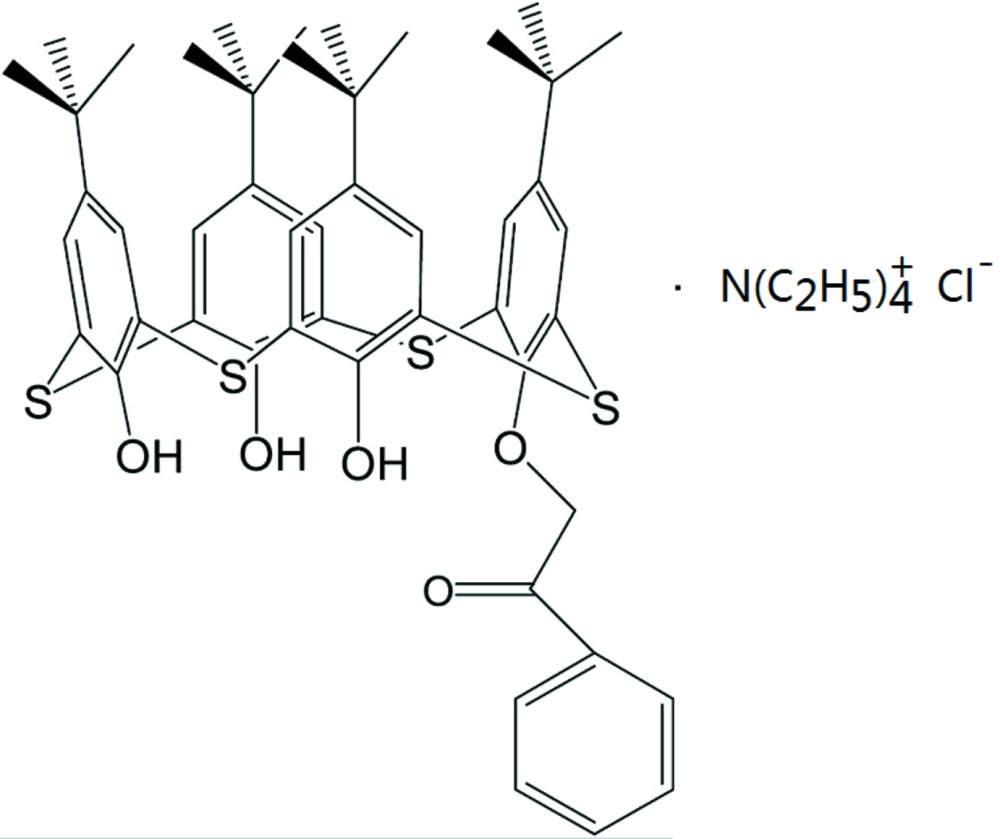


## Experimental   

### Crystal data  

C_48_H_54_O_5_S_4_·C_8_H_20_N^+^·Cl^−^
*M* *_r_* = 1004.85
Monoclinic, 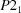

*a* = 19.4338 (6) Å
*b* = 14.2147 (3) Å
*c* = 20.8631 (6) Å
β = 103.184 (3)°
*V* = 5611.4 (3) Å^3^
*Z* = 4
Mo *K*α radiation
μ = 0.26 mm^−1^
*T* = 293 K
0.42 × 0.38 × 0.22 mm

### Data collection  

Agilent Xcalibur, Eos, Gemini diffractometer
Absorption correction: multi-scan (*CrysAlis PRO*; Agilent, 2014 ▸) *T*_min_ = 0.922, *T*_max_ = 1.000
40706 measured reflections
25992 independent reflections
18033 reflections with *I* > 2σ(*I*)
*R*_int_ = 0.035

### Refinement  

*R*[*F* ^2^ > 2σ(*F* ^2^)] = 0.079
*wR*(*F* ^2^) = 0.241
*S* = 1.05
25992 reflections
1208 parameters
8 restraints
H-atom parameters constrained
Δρ_max_ = 0.98 e Å^−3^
Δρ_min_ = −0.88 e Å^−3^
Absolute structure: Flack (1983 ▸)
Absolute structure parameter: 0.43 (8)

### 

Data collection: *CrysAlis PRO* (Agilent, 2014 ▸); cell refinement: *CrysAlis PRO*; data reduction: *CrysAlis PRO*; program(s) used to solve structure: *SHELXS2014* (Sheldrick, 2008 ▸); program(s) used to refine structure: *SHELXL2014* (Sheldrick, 2015 ▸); molecular graphics: *ORTEP-3 for Windows* (Farrugia, 2012 ▸); software used to prepare material for publication: *PLATON* (Spek, 2009 ▸).

## Supplementary Material

Crystal structure: contains datablock(s) global, I. DOI: 10.1107/S2056989015018617/xu5871sup1.cif

Structure factors: contains datablock(s) I. DOI: 10.1107/S2056989015018617/xu5871Isup2.hkl

**Fig. 1.**
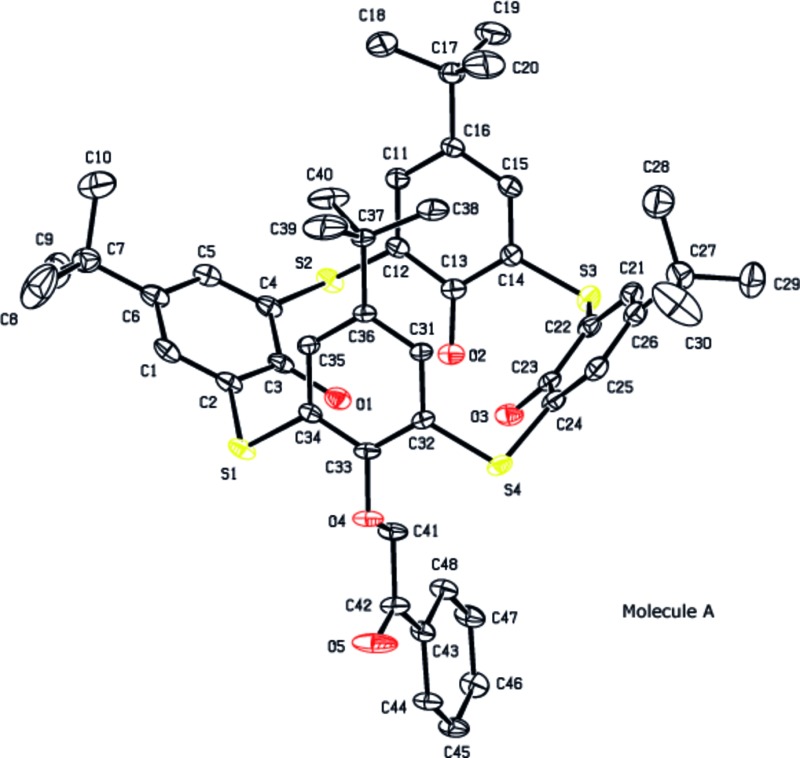
View of the molecule A of two molecules in the asymmetric unit with the atom-numbering scheme. Displacement ellipsoids for non-H atoms are drawn at the 30% probability level. All H atoms are omitted for clarity.

. DOI: 10.1107/S2056989015018617/xu5871fig1.tif
View of the mol­ecule A of two mol­ecules in the asymmetric unit with the atom-numbering scheme. Displacement ellipsoids for non-H atoms are drawn at the 30% probability level. All H atoms are omitted for clarity.

**Fig. 2.**
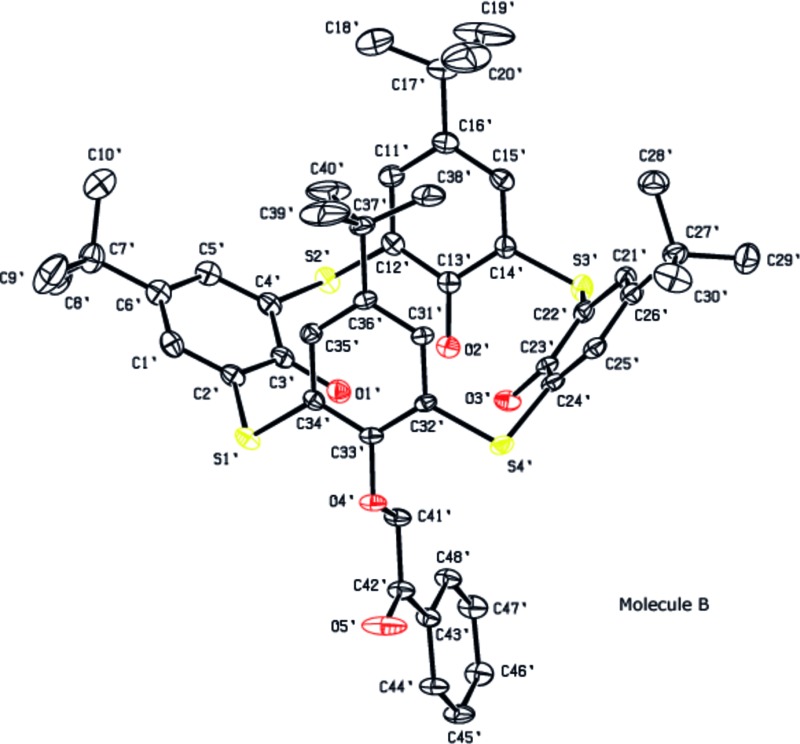
View of the molecule B of two molecules in the asymmetric unit with the atom-numbering scheme. Displacement ellipsoids for non-H atoms are drawn at the 30% probability level. All H atoms are omitted for clarity.

. DOI: 10.1107/S2056989015018617/xu5871fig2.tif
View of the mol­ecule B of two mol­ecules in the asymmetric unit with the atom-numbering scheme. Displacement ellipsoids for non-H atoms are drawn at the 30% probability level. All H atoms are omitted for clarity.

**Fig. 3.**
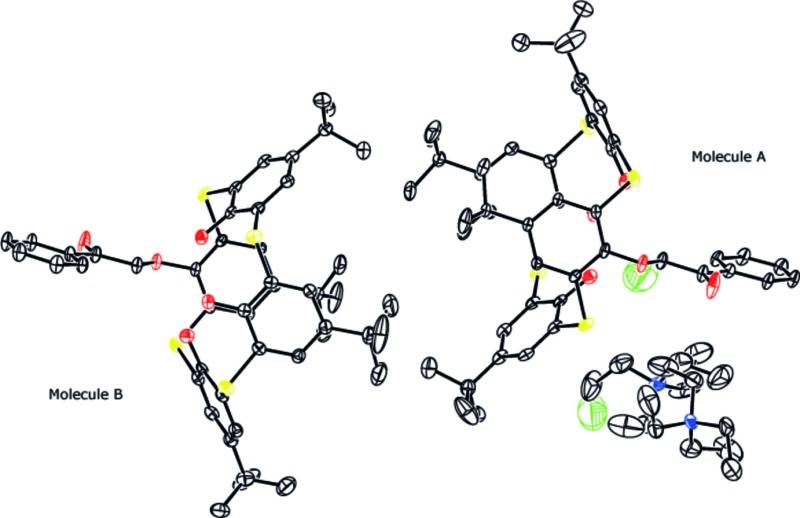
View of two molecules (A and B) with two solvent molecules in the asymmetric unit with the atom numbering scheme. Displacement ellipsoids for non-H atoms are drawn at the 30% probability level. All H atoms are omitted for clarity.

**Fig. 4.**
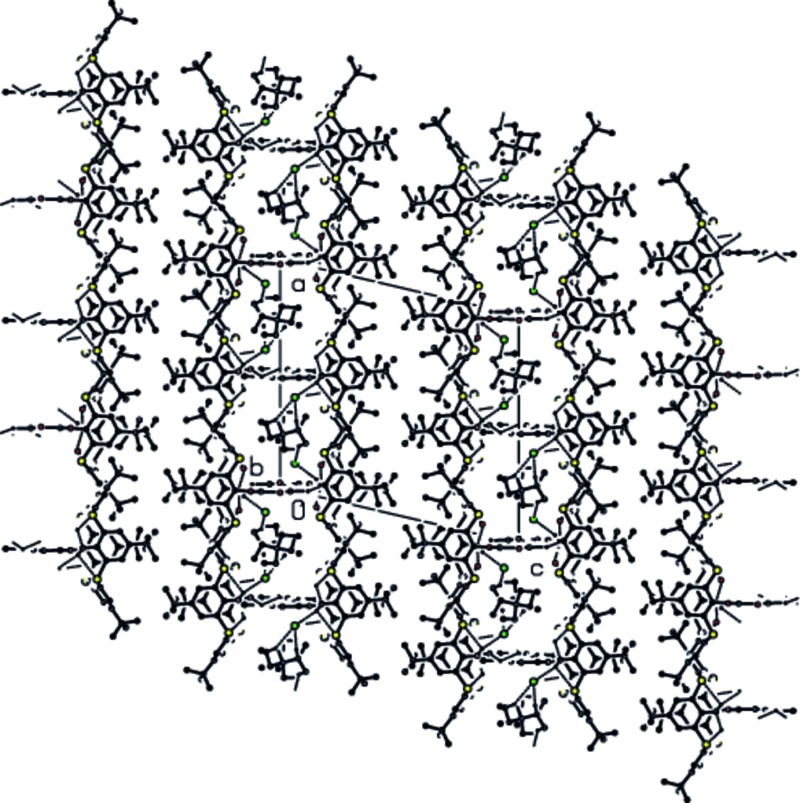
A view of the molecular packing down b axis. H atoms not involved in H bonding are omitted for clarity.

. DOI: 10.1107/S2056989015018617/xu5871fig3.tif
View of two mol­ecules (A and B) with two solvent mol­ecules in the asymmetric unit with the atom numbering scheme. Displacement ellipsoids for non-H atoms are drawn at the 30% probability level. All H atoms are omitted for clarity.

b . DOI: 10.1107/S2056989015018617/xu5871fig4.tif
A view of the mol­ecular packing down *b* axis. H atoms not involved in H bonding are omitted for clarity.

CCDC reference: 1429489

Additional supporting information:  crystallographic information; 3D view; checkCIF report

## Figures and Tables

**Table 1:** Hydrogen-bond geometry (, )

*D*H*A*	*D*H	H*A*	*D* *A*	*D*H*A*
O1H1*O*S2	0.82	2.61	3.110(4)	121
O1H1*O*O2	0.82	1.85	2.581(6)	147
O2H2*O*Cl2	0.82	2.30	3.048(10)	152
O3H3*O*S3	0.82	2.57	3.077(4)	121
O3H3*O*O2	0.82	1.91	2.567(6)	136
O1H6*O*S2	0.82	2.60	3.105(4)	121
O1H6*O*O2	0.82	1.85	2.580(5)	147
O1H6*O*Cl1^i^	0.82	2.81	3.338(11)	124
O2H7*O*Cl1^i^	0.82	2.27	3.006(10)	150
O3H8*O*S3	0.82	2.57	3.078(4)	121
O3H8*O*O2	0.82	1.90	2.567(5)	138
C103H10*L*Cl2	0.97	2.43	3.397(17)	174
C106H10*V*Cl2	0.96	2.64	3.382(19)	135
C202H20*I*O5	0.96	2.58	3.459(16)	152
C203H20*L*Cl1^ii^	0.97	2.41	3.373(15)	173
C204H20*P*Cl2^ii^	0.96	2.78	3.63(2)	147
C206H20*V*Cl1^ii^	0.96	2.55	3.353(18)	141
C41H41*A*O3	0.97	2.45	3.318(6)	149
C41H41*B*O1	0.97	2.46	3.253(6)	139
C41H41*C*S4	0.97	2.87	3.414(6)	116
C41H41*C*O3	0.97	2.45	3.313(6)	148
C41H41*D*O1	0.97	2.47	3.265(6)	139
C44H44O2^iii^	0.93	2.54	3.431(7)	160
C44H44O2^iv^	0.93	2.57	3.447(6)	158
C47H47O4^v^	0.93	2.55	3.368(6)	146
C47H47O5^v^	0.93	2.45	3.255(7)	145
C47H47O4^vi^	0.93	2.57	3.380(6)	146
C47H47O5^vi^	0.93	2.47	3.277(7)	145

Symmetry codes: (i) 
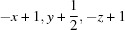
; (ii) 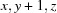; (iii) 
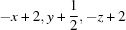
; (iv) 
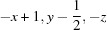
; (v) 
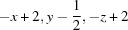
; (vi) 
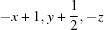
.

## supplementary crystallographic information

### S1. Comment

Calixarenes have been found to be an outstanding platform for creating attractive host molecules and have prominent host–guest recognition ability towards different ions. These macrocylic have been used for catalysis, molecular recognition or ion separtion and sensors (Gutsche, 1998; Shokova & Kovalev, 2003; Stoikov *et al.*, 2003). Since the discovery of *p*-*tert*-butylthiacalixarene in 1997 as a calixarene analogy with some additional features because of sulfur bridging atom in its skeleton structure, it attracts much attention in research rather than the classical calixarenes and exhibits a wide range of applications in supramolecular chemistry (Iki & Miyano, 2001; Morohashi *et al.*, 2002; Desroches *et al.*, 2003; Odo *et al.*, 2000; Odo *et al.*, 2001; Ben Ali *et al.*, 2001; Agrawal & Pancholi, 2007; Higuchi *et al.*, 2000; Omran & Antipin, 2014). Many calixarene derivatives are being used in both environmental and biomedical monitoring (McMahon *et al.*, 2003). Based on these findings we report here the synthesis and crystal structure of the title compound.

Two crystallographically independent molecules (A and B) in the asymmetric unit are shown in Figs 1 and 2, respectively, and together with two tetraethylammonium chloride molecules in Fig. 3. The bond lengths and bond angles of molecules A and B are within the normal ranges.

In the crystal structure, molecules are linked by C—H···O, C—H···S, C—H···Cl, O—H···O, O—H···S and O—H···Cl interactions, forming a three-dimensional network (Table 1). These lead to the formation of channels running parallel to the *a*-axis direction (Fig. 4).

### S2. Experimental

A mixture of *p*-*tert*-butylthiacalix[4]arene 1 g (1.38 mmol), anhydrous K_2_CO_3_ 5.0 g, TEAB 0.4 g and phenacyl bromide 0.28–0.55 g (1.38–2.76 mmol) in 50 ml benzene was heated at 373 K for 48 h. the mixture was filtered and the benzene layer was evaporated to dryness. The solid residue was washed by diluted hydrochloric acid solution, extracted with methylene chloride. The methylene chloride layer was dried over magnesium sulfate then filtered and concentrated. The residue was treated with methyl alcohol to deposit a white solid product. The product was filtered off, dried under vacuum and recrystalized from a mixture of methylene chloride/methanol (1*v*/1*v*) to give colourless crystals suitable for X-ray diffraction in 90% yield.

### S3. Refinement

All H atoms were positioned geometrically and constrained to ride on their parent atoms (C—H = 0.93, 0.96 and 0.97 Å, O—H = 0.82 Å). *U*_iso_(H) values were set to a multiple of *U*_eq_(C,*O*) with 1.5 for CH_3_ and OH, and 1.2 for CH and CH_2_. The (3 0 1), (4 0 8), (3 0 6), (−21 0 5), (1 4 0), (2 0 4), (−4 − 9 22), (−4 − 3 4), (1 0 8), (−12 5 3), (−2 0 10), (1 2 1), (−6 − 3 6), (−4 3 4), (1 − 4 0), (0 1 3), (3 0 0), (6 3 8) and (1 0 10) reflections were omitted owing to very bad agreement.

### Crystal data

**Table:**

C_48_H_54_O_5_S_4_·C_8_H_20_N^+^·Cl^−^	*F*(000) = 2152
*M**_r_* = 1004.85	*D*_x_ = 1.189 Mg m^−^^3^
Monoclinic, *P*2_1_	Mo *K*α radiation, λ = 0.71073 Å
Hall symbol: P 2yb	Cell parameters from 9238 reflections
*a* = 19.4338 (6) Å	θ = 3.2–31.7°
*b* = 14.2147 (3) Å	µ = 0.26 mm^−^^1^
*c* = 20.8631 (6) Å	*T* = 293 K
β = 103.184 (3)°	Prism, colourless
*V* = 5611.4 (3) Å^3^	0.42 × 0.38 × 0.22 mm
*Z* = 4	

### Data collection

**Table:**

Agilent Xcalibur, Eos, Gemini diffractometer	25992 independent reflections
Radiation source: Enhance (Mo) X-ray Source	18033 reflections with *I* > 2σ(*I*)
Graphite monochromator	*R*_int_ = 0.035
Detector resolution: 16.0416 pixels mm^-1^	θ_max_ = 32.8°, θ_min_ = 3.1°
ω scans	*h* = −27→27
Absorption correction: multi-scan (*CrysAlis PRO*; Agilent, 2014)	*k* = −19→21
*T*_min_ = 0.922, *T*_max_ = 1.000	*l* = −31→31
40706 measured reflections	

### Refinement

**Table:**

Refinement on *F*^2^	Hydrogen site location: inferred from neighbouring sites
Least-squares matrix: full	H-atom parameters constrained
*R*[*F*^2^ > 2σ(*F*^2^)] = 0.079	*w* = 1/[σ^2^(*F*_o_^2^) + (0.1278*P*)^2^ + 2.7781*P*] where *P* = (*F*_o_^2^ + 2*F*_c_^2^)/3
*wR*(*F*^2^) = 0.241	(Δ/σ)_max_ < 0.001
*S* = 1.05	Δρ_max_ = 0.98 e Å^−^^3^
25992 reflections	Δρ_min_ = −0.88 e Å^−^^3^
1208 parameters	Absolute structure: Flack (1983)
8 restraints	Absolute structure parameter: 0.43 (8)

### Special details

**Table:**

Geometry. Bond distances, angles *etc*. have been calculated using the rounded fractional coordinates. All su's are estimated from the variances of the (full) variance-covariance matrix. The cell e.s.d.'s are taken into account in the estimation of distances, angles and torsion angles
Refinement. Refinement on *F*^2^ for ALL reflections except those flagged by the user for potential systematic errors. Weighted *R*-factors *wR* and all goodnesses of fit *S* are based on *F*^2^, conventional *R*-factors *R* are based on *F*, with *F* set to zero for negative *F*^2^. The observed criterion of *F*^2^ > σ(*F*^2^) is used only for calculating -*R*-factor-obs *etc*. and is not relevant to the choice of reflections for refinement. *R*-factors based on *F*^2^ are statistically about twice as large as those based on *F*, and *R*-factors based on ALL data will be even larger.

### Fractional atomic coordinates and isotropic or equivalent isotropic displacement parameters (Å^2^)

**Table:**

	*x*	*y*	*z*	*U*_iso_*/*U*_eq_	
S1	0.80181 (8)	0.70396 (10)	0.81392 (7)	0.0378 (4)	
S2	0.79798 (8)	0.32086 (9)	0.76697 (7)	0.0375 (4)	
S3	1.08437 (7)	0.32100 (10)	0.78599 (7)	0.0386 (3)	
S4	1.08698 (7)	0.70419 (9)	0.83087 (6)	0.0352 (3)	
S1'	0.39116 (7)	0.32712 (10)	0.18651 (6)	0.0364 (3)	
S2'	0.41163 (7)	0.70921 (10)	0.23426 (7)	0.0376 (4)	
S3'	0.68815 (7)	0.70828 (10)	0.21467 (7)	0.0371 (3)	
S4'	0.66803 (7)	0.32534 (9)	0.16843 (6)	0.0336 (3)	
Cl1	0.5890 (5)	0.1924 (8)	0.9383 (4)	0.272 (5)	
O1	0.8589 (2)	0.5115 (3)	0.82787 (19)	0.0398 (11)	
O2	0.9501 (2)	0.3800 (3)	0.82638 (16)	0.0332 (10)	
O3	1.0475 (2)	0.5057 (3)	0.84746 (18)	0.0393 (11)	
O4	0.9539 (2)	0.7408 (2)	0.87704 (15)	0.0354 (9)	
O5	0.9786 (4)	0.7796 (3)	1.00075 (19)	0.071 (2)	
Cl2	0.8781 (5)	0.3360 (8)	0.9383 (4)	0.269 (5)	
C1	0.6904 (3)	0.6073 (4)	0.7405 (3)	0.0373 (17)	
O1'	0.4415 (2)	0.5191 (3)	0.17254 (19)	0.0386 (11)	
C2	0.7589 (3)	0.6001 (4)	0.7784 (3)	0.0345 (14)	
O2'	0.53388 (19)	0.6500 (2)	0.17442 (16)	0.0316 (10)	
C3	0.7947 (3)	0.5140 (4)	0.7881 (2)	0.0323 (14)	
O3'	0.6206 (2)	0.5239 (3)	0.15314 (17)	0.0370 (11)	
C4	0.7594 (3)	0.4353 (4)	0.7545 (2)	0.0336 (14)	
O4'	0.5115 (2)	0.2898 (2)	0.12228 (15)	0.0319 (9)	
C5	0.6911 (3)	0.4432 (4)	0.7169 (3)	0.0367 (16)	
O5'	0.4777 (4)	0.2509 (3)	−0.00141 (19)	0.072 (2)	
C6	0.6547 (3)	0.5280 (4)	0.7093 (3)	0.0399 (17)	
C7	0.5784 (4)	0.5339 (5)	0.6676 (3)	0.0467 (17)	
C8	0.5516 (6)	0.6335 (8)	0.6604 (7)	0.119 (5)	
C9	0.5321 (4)	0.4741 (8)	0.7010 (4)	0.075 (3)	
C10	0.5757 (6)	0.4932 (11)	0.5992 (5)	0.105 (5)	
C11	0.8604 (3)	0.2973 (4)	0.6625 (2)	0.0331 (12)	
C12	0.8709 (2)	0.3240 (4)	0.7288 (2)	0.0283 (11)	
C13	0.9400 (3)	0.3436 (3)	0.7676 (2)	0.0290 (14)	
C14	0.9963 (2)	0.3213 (3)	0.7379 (2)	0.0292 (11)	
C15	0.9840 (3)	0.2946 (3)	0.6716 (2)	0.0318 (14)	
C16	0.9163 (3)	0.2858 (4)	0.6318 (2)	0.0311 (12)	
C17	0.9045 (3)	0.2603 (4)	0.5588 (2)	0.0402 (16)	
C18	0.8299 (5)	0.2777 (9)	0.5210 (3)	0.091 (4)	
C19	0.9228 (5)	0.1576 (6)	0.5523 (4)	0.069 (3)	
C20	0.9535 (5)	0.3218 (8)	0.5270 (3)	0.083 (3)	
C21	1.1611 (3)	0.4491 (4)	0.7343 (3)	0.0384 (17)	
C22	1.1141 (3)	0.4370 (4)	0.7740 (2)	0.0349 (14)	
C23	1.0916 (3)	0.5131 (4)	0.8073 (2)	0.0301 (11)	
C24	1.1186 (3)	0.6025 (4)	0.7965 (2)	0.0351 (12)	
C25	1.1662 (3)	0.6138 (4)	0.7564 (3)	0.0375 (16)	
C26	1.1876 (3)	0.5385 (4)	0.7232 (3)	0.0394 (17)	
C27	1.2369 (4)	0.5485 (5)	0.6768 (3)	0.0490 (19)	
C28	1.1999 (5)	0.5128 (8)	0.6084 (4)	0.077 (3)	
C29	1.3035 (4)	0.4928 (8)	0.7018 (5)	0.077 (3)	
C30	1.2592 (8)	0.6506 (7)	0.6711 (7)	0.114 (6)	
C31	0.9866 (2)	0.6758 (3)	0.7159 (2)	0.0258 (11)	
C32	0.9990 (2)	0.7007 (3)	0.78212 (19)	0.0243 (10)	
C33	0.9425 (3)	0.7144 (3)	0.81181 (19)	0.0277 (13)	
C34	0.8743 (3)	0.7014 (4)	0.7749 (2)	0.0278 (11)	
C35	0.8624 (3)	0.6776 (4)	0.7084 (2)	0.0298 (11)	
C36	0.9189 (3)	0.6639 (3)	0.6779 (2)	0.0271 (11)	
C37	0.9057 (3)	0.6351 (4)	0.6048 (2)	0.0340 (13)	
C38	0.9711 (4)	0.5952 (9)	0.5879 (3)	0.084 (4)	
C39	0.8830 (6)	0.7202 (7)	0.5621 (3)	0.079 (3)	
C40	0.8473 (5)	0.5604 (7)	0.5886 (3)	0.070 (3)	
C41	0.9633 (3)	0.6610 (3)	0.9205 (2)	0.0348 (14)	
C42	0.9788 (3)	0.6964 (4)	0.9898 (2)	0.0351 (13)	
C43	0.9947 (3)	0.6261 (3)	1.0436 (2)	0.0312 (14)	
C44	1.0124 (3)	0.6590 (4)	1.1089 (2)	0.0360 (14)	
C45	1.0281 (4)	0.5972 (4)	1.1603 (2)	0.0417 (18)	
C46	1.0254 (4)	0.5011 (4)	1.1481 (3)	0.0433 (18)	
C47	1.0081 (4)	0.4679 (4)	1.0844 (3)	0.0424 (16)	
C48	0.9926 (3)	0.5301 (4)	1.0321 (2)	0.0365 (14)	
C1'	0.3177 (3)	0.4237 (4)	0.2627 (3)	0.0391 (16)	
C2'	0.3667 (3)	0.4301 (4)	0.2231 (3)	0.0346 (14)	
C3'	0.3975 (3)	0.5166 (4)	0.2133 (2)	0.0314 (12)	
C4'	0.3794 (3)	0.5954 (4)	0.2468 (3)	0.0344 (12)	
C5'	0.3303 (3)	0.5871 (4)	0.2864 (3)	0.0383 (17)	
C6'	0.2988 (3)	0.5021 (4)	0.2947 (3)	0.0400 (17)	
C7'	0.2441 (4)	0.4977 (5)	0.3373 (4)	0.052 (2)	
C8'	0.2295 (7)	0.3968 (7)	0.3541 (6)	0.102 (5)	
C9'	0.1775 (4)	0.5495 (8)	0.3007 (4)	0.073 (3)	
C10'	0.2704 (5)	0.5479 (8)	0.4020 (4)	0.082 (3)	
C11'	0.5250 (3)	0.7316 (4)	0.3387 (3)	0.0363 (16)	
C12'	0.5031 (3)	0.7055 (4)	0.2726 (2)	0.0312 (11)	
C13'	0.5528 (3)	0.6864 (3)	0.2336 (2)	0.0285 (12)	
C14'	0.6242 (2)	0.7080 (4)	0.2634 (2)	0.0298 (11)	
C15'	0.6439 (3)	0.7340 (4)	0.3291 (3)	0.0339 (12)	
C16'	0.5966 (3)	0.7429 (4)	0.3697 (3)	0.0368 (14)	
C17'	0.6204 (4)	0.7690 (5)	0.4425 (3)	0.0492 (18)	
C18'	0.5621 (6)	0.7607 (10)	0.4780 (4)	0.096 (4)	
C19'	0.6451 (10)	0.8685 (8)	0.4470 (5)	0.149 (7)	
C20'	0.6783 (6)	0.6990 (11)	0.4766 (4)	0.116 (5)	
C21'	0.7901 (3)	0.5798 (4)	0.2654 (3)	0.0337 (14)	
C22'	0.7226 (3)	0.5926 (4)	0.2258 (2)	0.0311 (12)	
C23'	0.6846 (3)	0.5165 (4)	0.1927 (2)	0.0283 (11)	
C24'	0.7168 (3)	0.4263 (4)	0.2027 (2)	0.0321 (14)	
C25'	0.7840 (3)	0.4154 (4)	0.2423 (2)	0.0332 (14)	
C26'	0.8225 (3)	0.4916 (4)	0.2757 (3)	0.0353 (16)	
C27'	0.8948 (3)	0.4798 (4)	0.3216 (3)	0.0407 (17)	
C28'	0.8932 (4)	0.5136 (6)	0.3901 (3)	0.057 (2)	
C29'	0.9488 (4)	0.5381 (7)	0.2947 (4)	0.063 (3)	
C30'	0.9196 (5)	0.3764 (6)	0.3288 (4)	0.067 (3)	
C31'	0.6250 (3)	0.3515 (3)	0.2838 (2)	0.0272 (11)	
C32'	0.6039 (2)	0.3282 (3)	0.2173 (2)	0.0254 (11)	
C33'	0.5331 (2)	0.3155 (3)	0.18780 (19)	0.0248 (10)	
C34'	0.4828 (2)	0.3283 (4)	0.2256 (2)	0.0277 (11)	
C35'	0.5043 (3)	0.3506 (4)	0.2918 (2)	0.0308 (11)	
C36'	0.5755 (3)	0.3630 (4)	0.3224 (2)	0.0292 (11)	
C37'	0.5979 (3)	0.3920 (4)	0.3949 (2)	0.0369 (15)	
C38'	0.6710 (5)	0.4311 (9)	0.4127 (4)	0.089 (4)	
C39'	0.5951 (8)	0.3079 (8)	0.4373 (3)	0.106 (5)	
C40'	0.5481 (5)	0.4694 (9)	0.4094 (4)	0.098 (4)	
C41'	0.5008 (3)	0.3695 (3)	0.0793 (2)	0.0317 (13)	
C42'	0.4819 (3)	0.3344 (4)	0.0099 (2)	0.0343 (14)	
C43'	0.4714 (3)	0.4048 (4)	−0.0438 (2)	0.0312 (14)	
C44'	0.4567 (3)	0.3710 (4)	−0.1090 (2)	0.0372 (14)	
C45'	0.4467 (4)	0.4330 (4)	−0.1606 (2)	0.0439 (18)	
C46'	0.4517 (4)	0.5298 (4)	−0.1485 (3)	0.0426 (18)	
C47'	0.4655 (3)	0.5623 (4)	−0.0849 (3)	0.0427 (18)	
C48'	0.4762 (3)	0.5000 (4)	−0.0325 (2)	0.0356 (14)	
N1	0.7316 (3)	0.5160 (5)	0.9884 (3)	0.0496 (17)	
C101	0.6644 (5)	0.5741 (8)	0.9824 (6)	0.091 (4)	
C102	0.6541 (8)	0.6476 (10)	0.9349 (10)	0.148 (8)	
C103	0.7320 (7)	0.4724 (9)	0.9252 (5)	0.098 (4)	
C104	0.6633 (9)	0.4157 (13)	0.8927 (6)	0.148 (8)	
C105	0.7357 (8)	0.4551 (14)	1.0466 (6)	0.179 (8)	
C106	0.7984 (10)	0.3894 (17)	1.0618 (7)	0.200 (12)	
C107	0.7949 (7)	0.5819 (12)	1.0075 (7)	0.119 (6)	
C108	0.8067 (9)	0.6162 (19)	1.0767 (9)	0.202 (12)	
N2	0.7630 (3)	1.0163 (4)	0.9884 (3)	0.0482 (16)	
C201	0.8276 (4)	0.9585 (7)	0.9832 (5)	0.073 (3)	
C202	0.8157 (7)	0.8812 (9)	0.9367 (9)	0.137 (7)	
C203	0.7296 (5)	1.0612 (8)	0.9248 (4)	0.080 (3)	
C204	0.7792 (8)	1.1205 (12)	0.8923 (6)	0.133 (6)	
C205	0.7912 (5)	1.0878 (9)	1.0404 (5)	0.107 (4)	
C206	0.7330 (8)	1.1501 (13)	1.0580 (7)	0.168 (8)	
C207	0.7100 (6)	0.9540 (11)	1.0089 (6)	0.103 (5)	
C208	0.7315 (7)	0.9154 (16)	1.0784 (8)	0.173 (8)	
H1	0.66790	0.66540	0.73570	0.0450*	
H1O	0.87440	0.45770	0.82950	0.0600*	
H2O	0.92590	0.35180	0.84760	0.0500*	
H3O	1.03590	0.45060	0.84950	0.0590*	
H5	0.66880	0.38990	0.69600	0.0440*	
H8A	0.55340	0.65960	0.70320	0.1780*	
H8B	0.50370	0.63410	0.63510	0.1780*	
H8C	0.58070	0.67030	0.63840	0.1780*	
H9A	0.53270	0.49950	0.74380	0.1120*	
H9B	0.54980	0.41080	0.70550	0.1120*	
H9C	0.48450	0.47420	0.67490	0.1120*	
H10A	0.59310	0.42970	0.60350	0.1580*	
H10B	0.60460	0.53060	0.57740	0.1580*	
H10C	0.52780	0.49360	0.57390	0.1580*	
H11	0.81460	0.28700	0.63830	0.0390*	
H15	1.02260	0.28220	0.65350	0.0380*	
H18A	0.81820	0.34290	0.52460	0.1360*	
H18B	0.79800	0.23940	0.53870	0.1360*	
H18C	0.82580	0.26210	0.47550	0.1360*	
H19A	0.97050	0.14630	0.57610	0.1030*	
H19B	0.91850	0.14270	0.50660	0.1030*	
H19C	0.89120	0.11880	0.56990	0.1030*	
H20A	1.00180	0.31200	0.54980	0.1240*	
H20B	0.94150	0.38690	0.53000	0.1240*	
H20C	0.94770	0.30470	0.48160	0.1240*	
H21	1.17580	0.39680	0.71420	0.0460*	
H25	1.18420	0.67340	0.75170	0.0450*	
H28A	1.23120	0.51910	0.57910	0.1160*	
H28B	1.15790	0.54920	0.59210	0.1160*	
H28C	1.18740	0.44790	0.61120	0.1160*	
H29A	1.32660	0.51480	0.74490	0.1150*	
H29B	1.33460	0.50070	0.67240	0.1150*	
H29C	1.29200	0.42740	0.70410	0.1150*	
H30A	1.28240	0.67360	0.71380	0.1710*	
H30B	1.21820	0.68830	0.65380	0.1710*	
H30C	1.29110	0.65410	0.64220	0.1710*	
H31	1.02490	0.66700	0.69680	0.0310*	
H35	0.81630	0.67080	0.68390	0.0360*	
H38A	0.98580	0.54010	0.61400	0.1270*	
H38B	1.00810	0.64130	0.59680	0.1270*	
H38C	0.96100	0.57870	0.54210	0.1270*	
H39A	0.91950	0.76720	0.57170	0.1180*	
H39B	0.84020	0.74520	0.57090	0.1180*	
H39C	0.87500	0.70250	0.51660	0.1180*	
H40A	0.86120	0.50600	0.61580	0.1050*	
H40B	0.83990	0.54290	0.54310	0.1050*	
H40C	0.80430	0.58550	0.59690	0.1050*	
H41A	1.00220	0.62250	0.91350	0.0420*	
H41B	0.92080	0.62290	0.91180	0.0420*	
H44	1.01340	0.72330	1.11710	0.0430*	
H45	1.04070	0.61940	1.20330	0.0500*	
H46	1.03520	0.45890	1.18310	0.0520*	
H47	1.00690	0.40340	1.07660	0.0510*	
H48	0.98070	0.50740	0.98910	0.0440*	
H1'	0.29730	0.36580	0.26770	0.0470*	
H8A'	0.21250	0.36190	0.31430	0.1520*	
H8B'	0.19460	0.39600	0.38000	0.1520*	
H8C'	0.27240	0.36860	0.37870	0.1520*	
H9A'	0.15910	0.51920	0.25920	0.1100*	
H5'	0.31870	0.64020	0.30770	0.0460*	
H9B'	0.18920	0.61360	0.29330	0.1100*	
H9C'	0.14270	0.54810	0.32670	0.1100*	
H6O	0.45610	0.57290	0.17070	0.0580*	
H7O	0.49830	0.67670	0.15380	0.0470*	
H8O	0.60770	0.57900	0.15160	0.0550*	
H10D	0.28040	0.61230	0.39390	0.1230*	
H10E	0.31270	0.51780	0.42610	0.1230*	
H10F	0.23490	0.54530	0.42730	0.1230*	
H11'	0.49110	0.74170	0.36300	0.0440*	
H15'	0.69140	0.74610	0.34700	0.0410*	
H18D	0.54540	0.69690	0.47550	0.1440*	
H18E	0.52400	0.80170	0.45800	0.1440*	
H18F	0.57930	0.77800	0.52330	0.1440*	
H19D	0.68290	0.87510	0.42460	0.2210*	
H19E	0.66170	0.88560	0.49240	0.2210*	
H19F	0.60670	0.90900	0.42680	0.2210*	
H20D	0.65950	0.63630	0.47240	0.1740*	
H20E	0.69360	0.71480	0.52240	0.1740*	
H20F	0.71770	0.70250	0.45610	0.1740*	
H21'	0.81460	0.63200	0.28580	0.0400*	
H25'	0.80430	0.35580	0.24690	0.0390*	
H28D	0.93920	0.50600	0.41870	0.0850*	
H28E	0.85940	0.47730	0.40670	0.0850*	
H28F	0.88000	0.57880	0.38840	0.0850*	
H29D	0.99470	0.53140	0.32330	0.0940*	
H29E	0.93510	0.60310	0.29240	0.0940*	
H29F	0.95010	0.51610	0.25140	0.0940*	
H30D	0.96540	0.37290	0.35820	0.1000*	
H30E	0.92220	0.35210	0.28650	0.1000*	
H30F	0.88650	0.33970	0.34620	0.1000*	
H31'	0.67280	0.35950	0.30290	0.0330*	
H35'	0.47040	0.35740	0.31650	0.0370*	
H38D	0.67360	0.48620	0.38670	0.1330*	
H38E	0.70380	0.38480	0.40420	0.1330*	
H38F	0.68270	0.44740	0.45850	0.1330*	
H39D	0.62640	0.26020	0.42780	0.1590*	
H39E	0.54770	0.28380	0.42840	0.1590*	
H39F	0.60950	0.32560	0.48280	0.1590*	
H40D	0.55090	0.52340	0.38240	0.1470*	
H40E	0.56190	0.48690	0.45500	0.1470*	
H40F	0.50040	0.44620	0.39980	0.1470*	
H41C	0.54360	0.40710	0.08630	0.0380*	
H41D	0.46300	0.40860	0.08800	0.0380*	
H44'	0.45370	0.30660	−0.11700	0.0440*	
H45'	0.43660	0.41080	−0.20360	0.0530*	
H46'	0.44580	0.57200	−0.18340	0.0510*	
H47'	0.46760	0.62670	−0.07690	0.0510*	
H48'	0.48660	0.52280	0.01040	0.0430*	
H1QW	0.76530	0.64880	1.08260	0.3040*	
H10G	0.62420	0.53190	0.97170	0.1090*	
H10H	0.66490	0.60160	1.02510	0.1090*	
H10I	0.61110	0.68050	0.93540	0.2220*	
H10J	0.65110	0.62130	0.89190	0.2220*	
H10K	0.69320	0.69060	0.94520	0.2220*	
H10L	0.77210	0.43010	0.93120	0.1180*	
H10M	0.73890	0.52130	0.89490	0.1180*	
H10N	0.66840	0.38950	0.85160	0.2220*	
H10O	0.62330	0.45720	0.88490	0.2220*	
H10P	0.65630	0.36590	0.92160	0.2220*	
H10Q	0.84640	0.65820	1.08570	0.3040*	
H10R	0.69280	0.41790	1.03990	0.2130*	
H10S	0.73720	0.49490	1.08470	0.2130*	
H10T	0.79570	0.35070	1.09880	0.2980*	
H10U	0.84120	0.42550	1.07190	0.2980*	
H10V	0.79800	0.35020	1.02420	0.2980*	
H10W	0.81600	0.56360	1.10630	0.3040*	
H10Y	0.83690	0.54920	1.00190	0.1430*	
H10Z	0.78760	0.63550	0.97800	0.1430*	
H2QW	0.77380	0.87880	1.08290	0.2600*	
H20G	0.86200	1.00060	0.97140	0.0880*	
H20H	0.84860	0.93320	1.02640	0.0880*	
H20I	0.85960	0.84940	0.93800	0.2050*	
H20J	0.79720	0.90520	0.89310	0.2050*	
H20K	0.78260	0.83790	0.94810	0.2050*	
H20L	0.69170	1.10150	0.93170	0.0960*	
H20M	0.70850	1.01230	0.89420	0.0960*	
H20N	0.75280	1.14580	0.85140	0.1990*	
H20O	0.81660	1.08140	0.88420	0.1990*	
H20P	0.79900	1.17110	0.92110	0.1990*	
H20Q	0.69430	0.87640	1.08700	0.2600*	
H20R	0.82480	1.12770	1.02540	0.1280*	
H20S	0.81640	1.05560	1.07980	0.1280*	
H20T	0.75410	1.19430	1.09160	0.2520*	
H20U	0.70000	1.11130	1.07380	0.2520*	
H20V	0.70870	1.18360	1.01950	0.2520*	
H20W	0.73990	0.96660	1.10920	0.2600*	
H20Y	0.70010	0.90150	0.97860	0.1230*	
H20Z	0.66640	0.98910	1.00480	0.1230*	

### Atomic displacement parameters (Å^2^)

**Table:**

	*U*^11^	*U*^22^	*U*^33^	*U*^12^	*U*^13^	*U*^23^
S1	0.0502 (7)	0.0276 (6)	0.0421 (6)	−0.0061 (6)	0.0242 (5)	−0.0121 (6)
S2	0.0481 (7)	0.0233 (5)	0.0469 (7)	−0.0079 (6)	0.0232 (6)	−0.0039 (6)
S3	0.0391 (6)	0.0228 (5)	0.0477 (7)	0.0044 (6)	−0.0029 (5)	0.0039 (6)
S4	0.0399 (6)	0.0267 (5)	0.0322 (5)	−0.0033 (5)	−0.0057 (4)	−0.0051 (5)
S1'	0.0349 (6)	0.0282 (6)	0.0407 (6)	−0.0020 (6)	−0.0024 (5)	−0.0047 (5)
S2'	0.0340 (6)	0.0250 (6)	0.0509 (7)	0.0050 (5)	0.0035 (5)	0.0017 (6)
S3'	0.0425 (6)	0.0236 (5)	0.0490 (7)	−0.0033 (6)	0.0182 (5)	0.0043 (6)
S4'	0.0453 (6)	0.0283 (6)	0.0307 (5)	−0.0011 (6)	0.0160 (5)	−0.0082 (5)
Cl1	0.269 (8)	0.315 (11)	0.201 (6)	0.065 (9)	−0.013 (6)	−0.056 (7)
O1	0.046 (2)	0.0318 (19)	0.0434 (19)	−0.0051 (18)	0.0139 (17)	−0.0103 (16)
O2	0.049 (2)	0.0238 (16)	0.0258 (15)	−0.0009 (16)	0.0066 (14)	0.0030 (13)
O3	0.053 (2)	0.0269 (17)	0.0362 (18)	−0.0054 (17)	0.0062 (16)	−0.0052 (15)
O4	0.066 (2)	0.0207 (15)	0.0177 (13)	−0.0040 (17)	0.0061 (14)	−0.0029 (12)
O5	0.158 (6)	0.0225 (19)	0.0256 (17)	0.005 (3)	0.008 (3)	−0.0005 (15)
Cl2	0.285 (9)	0.313 (11)	0.217 (6)	0.072 (9)	0.073 (6)	0.024 (7)
C1	0.048 (3)	0.031 (3)	0.042 (3)	0.000 (2)	0.029 (2)	−0.003 (2)
O1'	0.040 (2)	0.0313 (19)	0.045 (2)	−0.0028 (17)	0.0109 (16)	−0.0065 (16)
C2	0.045 (3)	0.029 (2)	0.036 (2)	−0.007 (2)	0.023 (2)	−0.008 (2)
O2'	0.0395 (18)	0.0229 (16)	0.0308 (16)	−0.0003 (15)	0.0046 (14)	0.0044 (13)
C3	0.041 (3)	0.031 (2)	0.032 (2)	−0.010 (2)	0.023 (2)	−0.0080 (19)
O3'	0.045 (2)	0.0293 (18)	0.0340 (17)	0.0031 (17)	0.0033 (15)	−0.0058 (15)
C4	0.043 (3)	0.028 (2)	0.036 (2)	−0.007 (2)	0.022 (2)	−0.005 (2)
O4'	0.052 (2)	0.0217 (15)	0.0186 (13)	−0.0006 (15)	0.0011 (13)	−0.0004 (12)
C5	0.049 (3)	0.030 (3)	0.036 (2)	−0.008 (2)	0.020 (2)	−0.005 (2)
O5'	0.155 (6)	0.026 (2)	0.0244 (17)	−0.008 (3)	0.000 (2)	−0.0017 (16)
C6	0.053 (3)	0.033 (3)	0.040 (3)	−0.008 (3)	0.024 (2)	−0.006 (2)
C7	0.054 (3)	0.040 (3)	0.045 (3)	−0.001 (3)	0.009 (3)	−0.005 (3)
C8	0.096 (8)	0.060 (6)	0.161 (11)	0.013 (6)	−0.051 (8)	−0.006 (7)
C9	0.049 (4)	0.103 (7)	0.074 (5)	−0.004 (5)	0.017 (4)	0.006 (5)
C10	0.086 (6)	0.162 (12)	0.062 (5)	−0.003 (8)	0.004 (5)	−0.043 (7)
C11	0.037 (2)	0.029 (2)	0.033 (2)	−0.002 (2)	0.0072 (18)	−0.0043 (19)
C12	0.037 (2)	0.0188 (19)	0.031 (2)	−0.003 (2)	0.0117 (17)	−0.0015 (18)
C13	0.044 (3)	0.016 (2)	0.0256 (19)	−0.0004 (18)	0.0050 (18)	0.0030 (15)
C14	0.035 (2)	0.0163 (18)	0.034 (2)	0.0021 (19)	0.0032 (17)	0.0014 (18)
C15	0.040 (3)	0.022 (2)	0.035 (2)	0.001 (2)	0.0122 (19)	−0.0015 (18)
C16	0.040 (2)	0.028 (2)	0.026 (2)	0.003 (2)	0.0091 (18)	−0.0025 (18)
C17	0.049 (3)	0.040 (3)	0.030 (2)	0.005 (3)	0.006 (2)	−0.004 (2)
C18	0.085 (6)	0.146 (10)	0.030 (3)	0.046 (6)	−0.009 (3)	−0.018 (4)
C19	0.097 (6)	0.059 (4)	0.046 (3)	0.017 (4)	0.008 (4)	−0.020 (3)
C20	0.114 (7)	0.101 (7)	0.039 (3)	−0.014 (7)	0.031 (4)	−0.001 (4)
C21	0.035 (3)	0.032 (3)	0.043 (3)	0.003 (2)	−0.002 (2)	−0.003 (2)
C22	0.038 (3)	0.025 (2)	0.034 (2)	0.003 (2)	−0.0076 (19)	0.0024 (19)
C23	0.033 (2)	0.026 (2)	0.0250 (19)	0.0000 (19)	−0.0063 (16)	−0.0010 (17)
C24	0.036 (2)	0.028 (2)	0.034 (2)	−0.002 (2)	−0.0070 (19)	−0.0021 (19)
C25	0.035 (3)	0.030 (2)	0.040 (3)	0.000 (2)	−0.007 (2)	0.000 (2)
C26	0.033 (3)	0.036 (3)	0.044 (3)	−0.002 (2)	−0.002 (2)	−0.002 (2)
C27	0.050 (3)	0.044 (3)	0.055 (4)	−0.001 (3)	0.016 (3)	−0.001 (3)
C28	0.064 (5)	0.112 (8)	0.056 (4)	0.007 (5)	0.015 (4)	0.005 (5)
C29	0.042 (4)	0.104 (7)	0.082 (5)	0.007 (5)	0.010 (4)	−0.003 (5)
C30	0.169 (12)	0.052 (5)	0.162 (11)	−0.030 (7)	0.122 (10)	−0.013 (6)
C31	0.032 (2)	0.026 (2)	0.0192 (17)	−0.0020 (18)	0.0052 (15)	−0.0003 (16)
C32	0.033 (2)	0.0168 (18)	0.0203 (16)	−0.0033 (18)	0.0005 (14)	0.0020 (15)
C33	0.049 (3)	0.0166 (19)	0.0166 (16)	−0.001 (2)	0.0056 (16)	−0.0005 (15)
C34	0.037 (2)	0.022 (2)	0.0267 (18)	−0.001 (2)	0.0123 (16)	−0.0022 (17)
C35	0.033 (2)	0.030 (2)	0.0246 (19)	0.001 (2)	0.0030 (16)	−0.0044 (17)
C36	0.036 (2)	0.025 (2)	0.0196 (17)	−0.0012 (19)	0.0047 (16)	−0.0021 (16)
C37	0.037 (2)	0.045 (3)	0.0186 (18)	0.000 (2)	0.0036 (17)	−0.0048 (19)
C38	0.069 (5)	0.147 (10)	0.035 (3)	0.012 (6)	0.007 (3)	−0.036 (5)
C39	0.137 (8)	0.071 (5)	0.025 (3)	−0.002 (6)	0.011 (4)	0.007 (3)
C40	0.091 (6)	0.080 (5)	0.041 (3)	−0.040 (5)	0.019 (4)	−0.026 (4)
C41	0.063 (3)	0.019 (2)	0.0212 (19)	0.000 (2)	0.007 (2)	−0.0022 (17)
C42	0.060 (3)	0.023 (2)	0.0207 (18)	−0.002 (2)	0.0056 (19)	−0.0012 (17)
C43	0.047 (3)	0.021 (2)	0.026 (2)	0.007 (2)	0.0092 (19)	0.0029 (17)
C44	0.061 (3)	0.026 (2)	0.0207 (19)	0.001 (2)	0.009 (2)	−0.0033 (18)
C45	0.070 (4)	0.034 (3)	0.021 (2)	0.007 (3)	0.010 (2)	0.001 (2)
C46	0.065 (4)	0.033 (3)	0.032 (2)	0.010 (3)	0.011 (2)	0.013 (2)
C47	0.072 (4)	0.021 (2)	0.035 (2)	0.008 (3)	0.014 (3)	0.001 (2)
C48	0.056 (3)	0.027 (2)	0.028 (2)	0.001 (2)	0.013 (2)	−0.0021 (19)
C1'	0.031 (2)	0.037 (3)	0.045 (3)	0.001 (2)	0.000 (2)	0.007 (2)
C2'	0.031 (2)	0.032 (3)	0.036 (2)	0.001 (2)	−0.0024 (19)	−0.001 (2)
C3'	0.024 (2)	0.032 (2)	0.033 (2)	0.0054 (19)	−0.0044 (16)	−0.0002 (19)
C4'	0.029 (2)	0.032 (2)	0.038 (2)	0.004 (2)	−0.0013 (18)	0.001 (2)
C5'	0.039 (3)	0.033 (3)	0.040 (3)	0.006 (2)	0.003 (2)	0.002 (2)
C6'	0.039 (3)	0.038 (3)	0.041 (3)	0.005 (2)	0.005 (2)	0.004 (2)
C7'	0.052 (4)	0.046 (3)	0.061 (4)	0.002 (3)	0.021 (3)	0.006 (3)
C8'	0.152 (10)	0.066 (6)	0.120 (8)	−0.014 (7)	0.100 (8)	−0.007 (6)
C9'	0.047 (4)	0.107 (7)	0.070 (5)	0.017 (5)	0.021 (3)	0.009 (5)
C10'	0.087 (6)	0.100 (7)	0.068 (5)	−0.008 (6)	0.037 (5)	−0.015 (5)
C11'	0.043 (3)	0.032 (3)	0.034 (2)	0.004 (2)	0.009 (2)	−0.002 (2)
C12'	0.033 (2)	0.0198 (19)	0.039 (2)	0.003 (2)	0.0045 (17)	0.001 (2)
C13'	0.037 (2)	0.016 (2)	0.032 (2)	0.0019 (18)	0.0066 (18)	0.0036 (16)
C14'	0.032 (2)	0.0191 (19)	0.039 (2)	0.000 (2)	0.0098 (17)	−0.0002 (19)
C15'	0.035 (2)	0.025 (2)	0.039 (2)	0.001 (2)	0.0029 (19)	−0.004 (2)
C16'	0.043 (3)	0.030 (2)	0.035 (2)	0.004 (2)	0.004 (2)	−0.002 (2)
C17'	0.061 (4)	0.049 (3)	0.033 (2)	0.003 (3)	0.001 (2)	−0.009 (3)
C18'	0.108 (7)	0.139 (10)	0.043 (4)	−0.016 (8)	0.020 (4)	−0.015 (5)
C19'	0.29 (2)	0.080 (7)	0.055 (5)	−0.045 (10)	−0.004 (8)	−0.028 (5)
C20'	0.103 (7)	0.171 (13)	0.056 (5)	0.055 (9)	−0.021 (5)	−0.019 (7)
C21'	0.034 (2)	0.033 (3)	0.036 (2)	−0.003 (2)	0.0121 (19)	−0.004 (2)
C22'	0.038 (2)	0.029 (2)	0.030 (2)	−0.001 (2)	0.0152 (19)	0.0038 (19)
C23'	0.038 (2)	0.029 (2)	0.0202 (17)	0.001 (2)	0.0113 (16)	−0.0022 (17)
C24'	0.048 (3)	0.027 (2)	0.026 (2)	−0.001 (2)	0.018 (2)	−0.0054 (18)
C25'	0.045 (3)	0.028 (2)	0.031 (2)	0.001 (2)	0.018 (2)	−0.0020 (19)
C26'	0.040 (3)	0.035 (3)	0.034 (2)	0.001 (2)	0.015 (2)	−0.003 (2)
C27'	0.041 (3)	0.043 (3)	0.039 (3)	0.003 (3)	0.011 (2)	−0.008 (2)
C28'	0.059 (4)	0.069 (5)	0.039 (3)	0.001 (4)	0.004 (3)	−0.005 (3)
C29'	0.046 (4)	0.085 (6)	0.060 (4)	−0.003 (4)	0.019 (3)	0.000 (4)
C30'	0.069 (5)	0.049 (4)	0.070 (5)	0.020 (4)	−0.011 (4)	−0.007 (4)
C31'	0.030 (2)	0.029 (2)	0.0218 (17)	0.0049 (18)	0.0041 (15)	−0.0021 (16)
C32'	0.036 (2)	0.0187 (18)	0.0228 (17)	0.0021 (19)	0.0094 (15)	−0.0025 (16)
C33'	0.040 (2)	0.0149 (18)	0.0179 (16)	0.0010 (18)	0.0031 (15)	0.0005 (15)
C34'	0.030 (2)	0.023 (2)	0.0277 (19)	−0.003 (2)	0.0015 (15)	0.0018 (18)
C35'	0.034 (2)	0.033 (2)	0.0255 (19)	−0.001 (2)	0.0068 (17)	0.0017 (18)
C36'	0.036 (2)	0.030 (2)	0.0212 (18)	0.004 (2)	0.0057 (17)	0.0015 (17)
C37'	0.043 (3)	0.047 (3)	0.0205 (19)	0.001 (3)	0.0068 (18)	−0.003 (2)
C38'	0.067 (5)	0.156 (11)	0.040 (3)	−0.018 (6)	0.004 (3)	−0.038 (5)
C39'	0.201 (13)	0.083 (7)	0.026 (3)	−0.013 (8)	0.012 (5)	0.008 (4)
C40'	0.079 (6)	0.138 (9)	0.066 (5)	0.040 (6)	−0.007 (4)	−0.066 (6)
C41'	0.052 (3)	0.019 (2)	0.0211 (18)	−0.002 (2)	0.0021 (18)	0.0002 (16)
C42'	0.053 (3)	0.024 (2)	0.0237 (19)	−0.002 (2)	0.0040 (19)	−0.0012 (17)
C43'	0.044 (3)	0.024 (2)	0.0228 (19)	−0.006 (2)	0.0019 (18)	0.0019 (17)
C44'	0.059 (3)	0.028 (2)	0.0201 (19)	−0.001 (2)	0.000 (2)	−0.0028 (18)
C45'	0.072 (4)	0.036 (3)	0.020 (2)	−0.005 (3)	0.003 (2)	0.001 (2)
C46'	0.064 (4)	0.030 (3)	0.032 (2)	−0.004 (3)	0.007 (2)	0.010 (2)
C47'	0.065 (4)	0.024 (2)	0.036 (3)	−0.002 (3)	0.005 (2)	0.003 (2)
C48'	0.050 (3)	0.027 (2)	0.027 (2)	−0.005 (2)	0.003 (2)	−0.0018 (19)
N1	0.051 (3)	0.059 (3)	0.047 (3)	0.001 (3)	0.028 (2)	0.017 (3)
C101	0.072 (6)	0.092 (7)	0.112 (8)	0.020 (6)	0.028 (5)	0.040 (6)
C102	0.119 (10)	0.093 (9)	0.248 (19)	0.028 (8)	0.076 (12)	0.085 (11)
C103	0.123 (9)	0.105 (8)	0.081 (6)	0.043 (7)	0.055 (6)	0.039 (6)
C104	0.158 (13)	0.199 (17)	0.094 (8)	−0.106 (13)	0.042 (8)	−0.058 (10)
C105	0.171 (12)	0.29 (2)	0.114 (9)	0.168 (14)	0.114 (9)	0.145 (12)
C106	0.202 (17)	0.31 (3)	0.110 (10)	0.180 (19)	0.083 (11)	0.110 (14)
C107	0.097 (8)	0.145 (12)	0.126 (9)	0.001 (9)	0.047 (7)	−0.010 (9)
C108	0.129 (12)	0.33 (3)	0.164 (13)	−0.081 (16)	0.067 (11)	−0.150 (18)
N2	0.034 (2)	0.058 (3)	0.046 (3)	−0.004 (2)	−0.0045 (19)	−0.017 (2)
C201	0.053 (4)	0.076 (5)	0.086 (5)	0.006 (4)	0.006 (4)	−0.035 (5)
C202	0.089 (8)	0.090 (8)	0.231 (16)	0.011 (7)	0.035 (9)	−0.077 (10)
C203	0.068 (5)	0.102 (7)	0.060 (4)	0.010 (5)	−0.005 (4)	−0.024 (5)
C204	0.125 (10)	0.161 (13)	0.097 (8)	−0.059 (10)	−0.006 (7)	0.051 (9)
C205	0.079 (6)	0.139 (10)	0.081 (6)	0.038 (6)	−0.028 (5)	−0.077 (7)
C206	0.113 (9)	0.219 (18)	0.140 (11)	0.091 (11)	−0.039 (8)	−0.127 (12)
C207	0.069 (6)	0.140 (11)	0.102 (8)	−0.016 (7)	0.026 (5)	−0.025 (8)
C208	0.089 (8)	0.29 (2)	0.141 (12)	0.002 (13)	0.028 (8)	0.129 (15)

### Geometric parameters (Å, º)

**Table:**

S1—C2	1.773 (6)	C11'—C16'	1.404 (9)
S1—C34	1.781 (6)	C12'—C13'	1.424 (8)
S2—C4	1.785 (6)	C13'—C14'	1.419 (7)
S2—C12	1.777 (4)	C14'—C15'	1.387 (7)
S3—C14	1.776 (4)	C15'—C16'	1.391 (9)
S3—C22	1.784 (6)	C16'—C17'	1.529 (9)
S4—C24	1.784 (6)	C17'—C20'	1.548 (15)
S4—C32	1.780 (4)	C17'—C19'	1.490 (14)
S1'—C34'	1.782 (4)	C17'—C18'	1.493 (13)
S1'—C2'	1.766 (6)	C21'—C22'	1.394 (8)
S2'—C12'	1.776 (6)	C21'—C26'	1.397 (8)
S2'—C4'	1.776 (6)	C22'—C23'	1.400 (8)
S3'—C14'	1.776 (4)	C23'—C24'	1.421 (8)
S3'—C22'	1.771 (6)	C24'—C25'	1.386 (8)
S4'—C24'	1.778 (6)	C25'—C26'	1.407 (8)
S4'—C32'	1.782 (4)	C26'—C27'	1.518 (9)
O1—C3	1.332 (7)	C27'—C30'	1.543 (10)
O2—C13	1.304 (5)	C27'—C28'	1.515 (9)
O3—C23	1.332 (7)	C27'—C29'	1.540 (10)
O4—C33	1.380 (5)	C31'—C32'	1.394 (6)
O4—C41	1.438 (5)	C31'—C36'	1.398 (8)
O5—C42	1.205 (7)	C32'—C33'	1.385 (6)
O1—H1O	0.8200	C33'—C34'	1.401 (6)
O2—H2O	0.8200	C34'—C35'	1.385 (6)
O3—H3O	0.8200	C35'—C36'	1.397 (8)
C1—C6	1.404 (8)	C36'—C37'	1.532 (6)
C1—C2	1.388 (9)	C37'—C38'	1.491 (12)
O1'—C3'	1.337 (7)	C37'—C39'	1.496 (11)
C2—C3	1.400 (8)	C37'—C40'	1.540 (13)
O2'—C13'	1.312 (5)	C41'—C42'	1.496 (6)
C3—C4	1.412 (8)	C42'—C43'	1.481 (7)
O3'—C23'	1.331 (7)	C43'—C48'	1.373 (8)
C4—C5	1.384 (8)	C43'—C44'	1.409 (6)
O4'—C33'	1.384 (5)	C44'—C45'	1.370 (7)
O4'—C41'	1.430 (5)	C45'—C46'	1.398 (8)
C5—C6	1.388 (8)	C46'—C47'	1.373 (9)
O5'—C42'	1.209 (7)	C47'—C48'	1.385 (8)
C6—C7	1.541 (10)	N1—C105	1.479 (17)
C7—C10	1.530 (13)	N1—C107	1.525 (17)
C7—C9	1.518 (12)	N1—C101	1.526 (12)
C7—C8	1.504 (14)	N1—C103	1.459 (13)
C11—C12	1.404 (6)	C1'—H1'	0.9300
C11—C16	1.391 (8)	C5'—H5'	0.9300
C12—C13	1.428 (7)	C8'—H8B'	0.9600
C13—C14	1.411 (7)	C8'—H8C'	0.9600
C14—C15	1.401 (6)	C8'—H8A'	0.9600
C15—C16	1.392 (8)	C9'—H9C'	0.9600
C16—C17	1.531 (6)	C9'—H9A'	0.9600
C17—C20	1.549 (11)	C9'—H9B'	0.9600
C17—C18	1.505 (11)	C10'—H10E	0.9600
C17—C19	1.516 (10)	C10'—H10F	0.9600
C21—C22	1.377 (8)	C10'—H10D	0.9600
C21—C26	1.410 (8)	C11'—H11'	0.9300
C22—C23	1.408 (8)	C15'—H15'	0.9300
C23—C24	1.412 (8)	C18'—H18D	0.9600
C24—C25	1.391 (8)	C18'—H18E	0.9600
C25—C26	1.389 (8)	C18'—H18F	0.9600
C26—C27	1.516 (9)	C19'—H19E	0.9600
C27—C28	1.531 (11)	C19'—H19F	0.9600
C27—C30	1.527 (13)	C19'—H19D	0.9600
C27—C29	1.505 (12)	C20'—H20D	0.9600
C31—C36	1.383 (7)	C20'—H20F	0.9600
C31—C32	1.393 (6)	C20'—H20E	0.9600
C32—C33	1.392 (7)	C21'—H21'	0.9300
C33—C34	1.385 (8)	C25'—H25'	0.9300
C34—C35	1.395 (6)	C28'—H28E	0.9600
C35—C36	1.403 (8)	C28'—H28F	0.9600
C36—C37	1.543 (6)	C28'—H28D	0.9600
C37—C38	1.505 (10)	C29'—H29D	0.9600
C37—C40	1.535 (11)	C29'—H29E	0.9600
C37—C39	1.507 (10)	C29'—H29F	0.9600
C41—C42	1.495 (6)	C30'—H30D	0.9600
C42—C43	1.482 (6)	C30'—H30E	0.9600
C43—C48	1.385 (7)	C30'—H30F	0.9600
C43—C44	1.407 (6)	C31'—H31'	0.9300
C44—C45	1.366 (7)	C35'—H35'	0.9300
C45—C46	1.388 (8)	C38'—H38D	0.9600
C46—C47	1.378 (9)	C38'—H38E	0.9600
C47—C48	1.383 (8)	C38'—H38F	0.9600
C1—H1	0.9300	C39'—H39F	0.9600
O1'—H6O	0.8200	C39'—H39D	0.9600
O2'—H7O	0.8200	C39'—H39E	0.9600
O3'—H8O	0.8200	C40'—H40E	0.9600
C5—H5	0.9300	C40'—H40F	0.9600
C8—H8A	0.9600	C40'—H40D	0.9600
C8—H8B	0.9600	C41'—H41C	0.9700
C8—H8C	0.9600	C41'—H41D	0.9700
C9—H9B	0.9600	C44'—H44'	0.9300
C9—H9A	0.9600	C45'—H45'	0.9300
C9—H9C	0.9600	C46'—H46'	0.9300
C10—H10C	0.9600	C47'—H47'	0.9300
C10—H10B	0.9600	C48'—H48'	0.9300
C10—H10A	0.9600	C101—C102	1.42 (2)
C11—H11	0.9300	C103—C104	1.57 (2)
C15—H15	0.9300	C105—C106	1.51 (3)
C18—H18B	0.9600	C107—C108	1.49 (2)
C18—H18A	0.9600	N2—C205	1.496 (13)
C18—H18C	0.9600	N2—C207	1.493 (15)
C19—H19B	0.9600	N2—C201	1.525 (11)
C19—H19C	0.9600	N2—C203	1.482 (11)
C19—H19A	0.9600	C101—H10G	0.9700
C20—H20B	0.9600	C101—H10H	0.9700
C20—H20A	0.9600	C102—H10J	0.9600
C20—H20C	0.9600	C102—H10K	0.9600
C21—H21	0.9300	C102—H10I	0.9600
C25—H25	0.9300	C103—H10L	0.9700
C28—H28A	0.9600	C103—H10M	0.9700
C28—H28C	0.9600	C104—H10P	0.9600
C28—H28B	0.9600	C104—H10N	0.9600
C29—H29B	0.9600	C104—H10O	0.9600
C29—H29C	0.9600	C105—H10S	0.9700
C29—H29A	0.9600	C105—H10R	0.9700
C30—H30C	0.9600	C106—H10T	0.9600
C30—H30B	0.9600	C106—H10U	0.9600
C30—H30A	0.9600	C106—H10V	0.9600
C31—H31	0.9300	C107—H10Y	0.9700
C35—H35	0.9300	C107—H10Z	0.9700
C38—H38C	0.9600	C108—H10Q	0.9600
C38—H38A	0.9600	C108—H10W	0.9600
C38—H38B	0.9600	C108—H1QW	0.9600
C39—H39C	0.9600	C201—C202	1.449 (18)
C39—H39B	0.9600	C203—C204	1.548 (19)
C39—H39A	0.9600	C205—C206	1.55 (2)
C40—H40B	0.9600	C207—C208	1.52 (2)
C40—H40A	0.9600	C201—H20G	0.9700
C40—H40C	0.9600	C201—H20H	0.9700
C41—H41B	0.9700	C202—H20I	0.9600
C41—H41A	0.9700	C202—H20J	0.9600
C44—H44	0.9300	C202—H20K	0.9600
C45—H45	0.9300	C203—H20L	0.9700
C46—H46	0.9300	C203—H20M	0.9700
C47—H47	0.9300	C204—H20N	0.9600
C48—H48	0.9300	C204—H20O	0.9600
C1'—C2'	1.399 (9)	C204—H20P	0.9600
C1'—C6'	1.391 (8)	C205—H20R	0.9700
C2'—C3'	1.403 (8)	C205—H20S	0.9700
C3'—C4'	1.407 (8)	C206—H20T	0.9600
C4'—C5'	1.402 (9)	C206—H20U	0.9600
C5'—C6'	1.383 (8)	C206—H20V	0.9600
C6'—C7'	1.535 (10)	C207—H20Y	0.9700
C7'—C8'	1.519 (13)	C207—H20Z	0.9700
C7'—C9'	1.533 (12)	C208—H2QW	0.9600
C7'—C10'	1.509 (12)	C208—H20Q	0.9600
C11'—C12'	1.397 (7)	C208—H20W	0.9600
			
C2—S1—C34	97.9 (3)	C22'—C23'—C24'	117.4 (5)
C4—S2—C12	105.3 (3)	C23'—C24'—C25'	120.8 (5)
C14—S3—C22	102.7 (2)	S4'—C24'—C25'	119.5 (4)
C24—S4—C32	97.0 (2)	S4'—C24'—C23'	119.5 (4)
C2'—S1'—C34'	97.7 (3)	C24'—C25'—C26'	122.1 (5)
C4'—S2'—C12'	104.9 (3)	C21'—C26'—C25'	116.4 (5)
C14'—S3'—C22'	102.5 (3)	C21'—C26'—C27'	121.1 (5)
C24'—S4'—C32'	97.5 (2)	C25'—C26'—C27'	122.6 (5)
C33—O4—C41	112.1 (3)	C28'—C27'—C29'	109.6 (6)
C3—O1—H1O	110.00	C28'—C27'—C30'	106.5 (6)
C13—O2—H2O	109.00	C29'—C27'—C30'	108.9 (6)
C23—O3—H3O	109.00	C26'—C27'—C30'	113.0 (5)
C2—C1—C6	120.9 (5)	C26'—C27'—C28'	110.2 (5)
S1—C2—C3	119.8 (4)	C26'—C27'—C29'	108.7 (5)
C1—C2—C3	121.8 (5)	C32'—C31'—C36'	121.1 (5)
S1—C2—C1	118.4 (4)	C31'—C32'—C33'	120.7 (4)
O1—C3—C4	124.6 (5)	S4'—C32'—C33'	119.8 (3)
O1—C3—C2	118.5 (5)	S4'—C32'—C31'	119.3 (4)
C2—C3—C4	116.9 (5)	C32'—C33'—C34'	118.9 (4)
S2—C4—C3	120.6 (4)	O4'—C33'—C34'	119.8 (4)
C3—C4—C5	120.8 (5)	O4'—C33'—C32'	121.3 (4)
S2—C4—C5	118.2 (4)	S1'—C34'—C33'	119.6 (3)
C33'—O4'—C41'	112.2 (3)	S1'—C34'—C35'	120.1 (4)
C4—C5—C6	122.2 (5)	C33'—C34'—C35'	120.0 (4)
C5—C6—C7	120.8 (5)	C34'—C35'—C36'	121.8 (5)
C1—C6—C5	117.3 (5)	C35'—C36'—C37'	120.9 (5)
C1—C6—C7	121.9 (5)	C31'—C36'—C35'	117.5 (4)
C8—C7—C9	110.2 (8)	C31'—C36'—C37'	121.5 (5)
C6—C7—C9	107.8 (5)	C36'—C37'—C38'	112.7 (5)
C6—C7—C10	109.3 (7)	C36'—C37'—C39'	109.2 (5)
C8—C7—C10	109.0 (9)	C36'—C37'—C40'	109.7 (5)
C9—C7—C10	108.6 (8)	C39'—C37'—C40'	110.2 (7)
C6—C7—C8	112.0 (7)	C38'—C37'—C39'	108.2 (8)
C12—C11—C16	122.2 (5)	C38'—C37'—C40'	106.9 (7)
S2—C12—C11	118.9 (4)	O4'—C41'—C42'	108.1 (4)
S2—C12—C13	119.6 (3)	O5'—C42'—C41'	120.5 (4)
C11—C12—C13	121.2 (4)	O5'—C42'—C43'	121.6 (4)
O2—C13—C14	122.5 (5)	C41'—C42'—C43'	117.9 (4)
O2—C13—C12	122.1 (5)	C42'—C43'—C44'	117.5 (5)
C12—C13—C14	115.5 (4)	C42'—C43'—C48'	122.9 (4)
C13—C14—C15	121.3 (4)	C44'—C43'—C48'	119.5 (4)
S3—C14—C13	119.8 (3)	C43'—C44'—C45'	120.0 (5)
S3—C14—C15	118.9 (4)	C44'—C45'—C46'	120.0 (4)
C14—C15—C16	122.6 (5)	C45'—C46'—C47'	119.7 (5)
C11—C16—C15	116.5 (4)	C46'—C47'—C48'	120.6 (5)
C15—C16—C17	121.4 (5)	C43'—C48'—C47'	120.2 (4)
C11—C16—C17	122.0 (5)	C105—N1—C107	104.8 (9)
C16—C17—C19	109.4 (5)	C101—N1—C107	108.3 (8)
C16—C17—C20	109.0 (5)	C101—N1—C103	109.7 (8)
C18—C17—C20	107.2 (6)	C101—N1—C105	105.6 (9)
C16—C17—C18	113.0 (5)	C103—N1—C105	118.9 (10)
C19—C17—C20	109.0 (6)	C103—N1—C107	109.1 (8)
C18—C17—C19	109.2 (7)	C6'—C1'—H1'	119.00
C22—C21—C26	121.9 (5)	C2'—C1'—H1'	119.00
C21—C22—C23	121.6 (5)	C4'—C5'—H5'	119.00
S3—C22—C21	118.7 (4)	C6'—C5'—H5'	119.00
S3—C22—C23	119.6 (4)	H8A'—C8'—H8C'	109.00
O3—C23—C24	119.1 (5)	C7'—C8'—H8A'	110.00
O3—C23—C22	124.5 (5)	C7'—C8'—H8B'	110.00
C22—C23—C24	116.4 (5)	C7'—C8'—H8C'	109.00
S4—C24—C23	119.4 (4)	H8A'—C8'—H8B'	109.00
C23—C24—C25	121.5 (5)	H8B'—C8'—H8C'	109.00
S4—C24—C25	119.1 (4)	H9A'—C9'—H9C'	110.00
C24—C25—C26	121.8 (5)	C7'—C9'—H9B'	109.00
C21—C26—C25	116.8 (5)	H9B'—C9'—H9C'	110.00
C21—C26—C27	119.8 (5)	C7'—C9'—H9A'	109.00
C25—C26—C27	123.4 (5)	C7'—C9'—H9C'	109.00
C26—C27—C28	109.5 (6)	H9A'—C9'—H9B'	110.00
C26—C27—C29	110.4 (6)	C7'—C10'—H10E	109.00
C26—C27—C30	111.8 (7)	C7'—C10'—H10D	110.00
C28—C27—C29	109.4 (7)	H10D—C10'—H10F	109.00
C28—C27—C30	108.9 (8)	C7'—C10'—H10F	110.00
C29—C27—C30	106.9 (8)	H10D—C10'—H10E	109.00
C32—C31—C36	121.8 (4)	H10E—C10'—H10F	109.00
S4—C32—C33	119.6 (3)	C16'—C11'—H11'	119.00
S4—C32—C31	120.0 (3)	C12'—C11'—H11'	119.00
C31—C32—C33	120.1 (4)	C14'—C15'—H15'	118.00
O4—C33—C34	120.2 (5)	C16'—C15'—H15'	118.00
C32—C33—C34	119.1 (4)	H18E—C18'—H18F	109.00
O4—C33—C32	120.8 (4)	C17'—C18'—H18E	110.00
S1—C34—C33	120.0 (3)	C17'—C18'—H18D	109.00
C33—C34—C35	120.4 (5)	H18D—C18'—H18F	109.00
S1—C34—C35	119.4 (4)	C17'—C18'—H18F	110.00
C34—C35—C36	121.0 (5)	H18D—C18'—H18E	109.00
C35—C36—C37	121.0 (5)	C17'—C19'—H19D	109.00
C31—C36—C37	121.4 (5)	H19D—C19'—H19E	109.00
C31—C36—C35	117.6 (4)	C17'—C19'—H19E	109.00
C36—C37—C40	110.4 (4)	C17'—C19'—H19F	110.00
C39—C37—C40	108.9 (6)	H19D—C19'—H19F	109.00
C36—C37—C39	109.4 (5)	H19E—C19'—H19F	110.00
C38—C37—C39	108.2 (6)	C17'—C20'—H20E	110.00
C38—C37—C40	108.0 (7)	C17'—C20'—H20D	109.00
C36—C37—C38	111.9 (5)	H20E—C20'—H20F	110.00
O4—C41—C42	108.2 (4)	C17'—C20'—H20F	109.00
O5—C42—C43	121.8 (4)	H20D—C20'—H20E	109.00
O5—C42—C41	120.3 (4)	H20D—C20'—H20F	109.00
C41—C42—C43	117.8 (4)	C22'—C21'—H21'	119.00
C42—C43—C44	118.2 (4)	C26'—C21'—H21'	119.00
C42—C43—C48	122.7 (4)	C24'—C25'—H25'	119.00
C44—C43—C48	119.1 (4)	C26'—C25'—H25'	119.00
C43—C44—C45	120.5 (5)	C27'—C28'—H28E	109.00
C44—C45—C46	119.8 (5)	C27'—C28'—H28D	109.00
C45—C46—C47	120.3 (5)	H28D—C28'—H28F	110.00
C46—C47—C48	120.2 (5)	C27'—C28'—H28F	110.00
C43—C48—C47	120.1 (4)	H28D—C28'—H28E	109.00
C6—C1—H1	120.00	H28E—C28'—H28F	109.00
C2—C1—H1	120.00	C27'—C29'—H29F	109.00
C3'—O1'—H6O	109.00	C27'—C29'—H29D	110.00
C13'—O2'—H7O	109.00	C27'—C29'—H29E	109.00
C23'—O3'—H8O	109.00	H29D—C29'—H29F	109.00
C4—C5—H5	119.00	H29E—C29'—H29F	109.00
C6—C5—H5	119.00	H29D—C29'—H29E	110.00
H8A—C8—H8C	109.00	H30D—C30'—H30E	109.00
C7—C8—H8A	109.00	H30D—C30'—H30F	109.00
H8A—C8—H8B	109.00	C27'—C30'—H30D	110.00
H8B—C8—H8C	110.00	C27'—C30'—H30E	110.00
C7—C8—H8B	109.00	C27'—C30'—H30F	109.00
C7—C8—H8C	109.00	H30E—C30'—H30F	109.00
H9B—C9—H9C	110.00	C36'—C31'—H31'	119.00
C7—C9—H9C	110.00	C32'—C31'—H31'	120.00
C7—C9—H9B	109.00	C34'—C35'—H35'	119.00
H9A—C9—H9C	109.00	C36'—C35'—H35'	119.00
H9A—C9—H9B	109.00	C37'—C38'—H38E	109.00
C7—C9—H9A	109.00	C37'—C38'—H38D	109.00
H10A—C10—H10C	109.00	C37'—C38'—H38F	110.00
C7—C10—H10C	109.00	H38D—C38'—H38E	109.00
C7—C10—H10B	109.00	H38D—C38'—H38F	110.00
C7—C10—H10A	109.00	H38E—C38'—H38F	109.00
H10B—C10—H10C	110.00	H39D—C39'—H39E	109.00
H10A—C10—H10B	109.00	H39D—C39'—H39F	109.00
C12—C11—H11	119.00	C37'—C39'—H39F	109.00
C16—C11—H11	119.00	C37'—C39'—H39D	109.00
C14—C15—H15	119.00	C37'—C39'—H39E	109.00
C16—C15—H15	119.00	H39E—C39'—H39F	110.00
C17—C18—H18B	110.00	H40E—C40'—H40F	109.00
H18B—C18—H18C	110.00	C37'—C40'—H40D	109.00
H18A—C18—H18C	109.00	C37'—C40'—H40E	109.00
C17—C18—H18A	110.00	C37'—C40'—H40F	109.00
C17—C18—H18C	109.00	H40D—C40'—H40E	109.00
H18A—C18—H18B	109.00	H40D—C40'—H40F	109.00
C17—C19—H19B	109.00	O4'—C41'—H41D	110.00
C17—C19—H19A	109.00	C42'—C41'—H41C	110.00
H19A—C19—H19B	109.00	O4'—C41'—H41C	110.00
H19A—C19—H19C	109.00	H41C—C41'—H41D	108.00
C17—C19—H19C	110.00	C42'—C41'—H41D	110.00
H19B—C19—H19C	110.00	C43'—C44'—H44'	120.00
H20B—C20—H20C	109.00	C45'—C44'—H44'	120.00
C17—C20—H20C	110.00	C44'—C45'—H45'	120.00
C17—C20—H20B	110.00	C46'—C45'—H45'	120.00
C17—C20—H20A	109.00	C47'—C46'—H46'	120.00
H20A—C20—H20C	109.00	C45'—C46'—H46'	120.00
H20A—C20—H20B	109.00	C46'—C47'—H47'	120.00
C22—C21—H21	119.00	C48'—C47'—H47'	120.00
C26—C21—H21	119.00	C47'—C48'—H48'	120.00
C26—C25—H25	119.00	C43'—C48'—H48'	120.00
C24—C25—H25	119.00	N1—C101—C102	115.9 (10)
C27—C28—H28B	109.00	N1—C103—C104	115.3 (10)
H28A—C28—H28C	110.00	N1—C105—C106	115.1 (12)
C27—C28—H28A	109.00	N1—C107—C108	113.0 (12)
C27—C28—H28C	109.00	C201—N2—C207	109.5 (7)
H28A—C28—H28B	110.00	C203—N2—C205	111.7 (7)
H28B—C28—H28C	109.00	C203—N2—C207	108.8 (7)
C27—C29—H29C	110.00	C205—N2—C207	111.0 (7)
C27—C29—H29B	109.00	C201—N2—C205	104.3 (7)
H29A—C29—H29C	109.00	C201—N2—C203	111.6 (7)
H29A—C29—H29B	109.00	N1—C101—H10G	108.00
H29B—C29—H29C	109.00	H10G—C101—H10H	107.00
C27—C29—H29A	110.00	C102—C101—H10H	108.00
H30A—C30—H30C	109.00	N1—C101—H10H	108.00
H30A—C30—H30B	109.00	C102—C101—H10G	108.00
C27—C30—H30B	110.00	C101—C102—H10I	110.00
C27—C30—H30C	109.00	H10I—C102—H10J	109.00
C27—C30—H30A	109.00	C101—C102—H10J	109.00
H30B—C30—H30C	110.00	C101—C102—H10K	109.00
C36—C31—H31	119.00	H10I—C102—H10K	110.00
C32—C31—H31	119.00	H10J—C102—H10K	109.00
C36—C35—H35	119.00	N1—C103—H10M	108.00
C34—C35—H35	119.00	N1—C103—H10L	108.00
C37—C38—H38B	109.00	C104—C103—H10L	108.00
H38A—C38—H38C	109.00	C104—C103—H10M	108.00
C37—C38—H38A	109.00	H10L—C103—H10M	108.00
C37—C38—H38C	109.00	H10N—C104—H10O	109.00
H38A—C38—H38B	110.00	H10N—C104—H10P	109.00
H38B—C38—H38C	109.00	H10O—C104—H10P	110.00
H39A—C39—H39C	109.00	C103—C104—H10N	109.00
H39A—C39—H39B	109.00	C103—C104—H10O	109.00
C37—C39—H39C	109.00	C103—C104—H10P	110.00
C37—C39—H39A	109.00	C106—C105—H10R	109.00
H39B—C39—H39C	110.00	N1—C105—H10R	108.00
C37—C39—H39B	109.00	N1—C105—H10S	108.00
C37—C40—H40A	109.00	C106—C105—H10S	108.00
C37—C40—H40C	109.00	H10R—C105—H10S	107.00
H40A—C40—H40B	110.00	C105—C106—H10U	109.00
C37—C40—H40B	109.00	C105—C106—H10T	110.00
H40B—C40—H40C	110.00	H10T—C106—H10U	110.00
H40A—C40—H40C	109.00	H10T—C106—H10V	109.00
C42—C41—H41A	110.00	C105—C106—H10V	109.00
O4—C41—H41A	110.00	H10U—C106—H10V	110.00
H41A—C41—H41B	108.00	C108—C107—H10Y	109.00
C42—C41—H41B	110.00	C108—C107—H10Z	109.00
O4—C41—H41B	110.00	N1—C107—H10Y	109.00
C43—C44—H44	120.00	N1—C107—H10Z	109.00
C45—C44—H44	120.00	H10Y—C107—H10Z	108.00
C46—C45—H45	120.00	H10Q—C108—H10W	109.00
C44—C45—H45	120.00	C107—C108—H1QW	109.00
C45—C46—H46	120.00	C107—C108—H10Q	109.00
C47—C46—H46	120.00	C107—C108—H10W	109.00
C46—C47—H47	120.00	H1QW—C108—H10Q	110.00
C48—C47—H47	120.00	H1QW—C108—H10W	109.00
C47—C48—H48	120.00	N2—C201—C202	116.6 (8)
C43—C48—H48	120.00	N2—C203—C204	115.9 (8)
C2'—C1'—C6'	121.4 (5)	N2—C205—C206	113.2 (9)
C1'—C2'—C3'	120.9 (5)	N2—C207—C208	115.5 (10)
S1'—C2'—C3'	119.9 (4)	N2—C201—H20G	108.00
S1'—C2'—C1'	119.2 (4)	N2—C201—H20H	108.00
O1'—C3'—C2'	118.2 (5)	C202—C201—H20G	108.00
O1'—C3'—C4'	124.3 (5)	C202—C201—H20H	108.00
C2'—C3'—C4'	117.5 (5)	H20G—C201—H20H	107.00
C3'—C4'—C5'	120.4 (5)	C201—C202—H20I	109.00
S2'—C4'—C3'	121.0 (4)	C201—C202—H20J	109.00
S2'—C4'—C5'	118.3 (4)	C201—C202—H20K	110.00
C4'—C5'—C6'	121.8 (5)	H20I—C202—H20J	109.00
C1'—C6'—C5'	117.8 (6)	H20I—C202—H20K	109.00
C1'—C6'—C7'	122.6 (5)	H20J—C202—H20K	110.00
C5'—C6'—C7'	119.5 (5)	N2—C203—H20L	108.00
C8'—C7'—C9'	112.8 (8)	N2—C203—H20M	108.00
C8'—C7'—C10'	106.5 (8)	C204—C203—H20L	108.00
C9'—C7'—C10'	107.1 (7)	C204—C203—H20M	108.00
C6'—C7'—C9'	108.0 (6)	H20L—C203—H20M	107.00
C6'—C7'—C10'	111.1 (7)	C203—C204—H20N	109.00
C6'—C7'—C8'	111.3 (7)	C203—C204—H20O	110.00
C12'—C11'—C16'	122.1 (6)	C203—C204—H20P	110.00
S2'—C12'—C11'	119.0 (4)	H20N—C204—H20O	109.00
S2'—C12'—C13'	119.2 (3)	H20N—C204—H20P	109.00
C11'—C12'—C13'	121.4 (5)	H20O—C204—H20P	109.00
O2'—C13'—C14'	122.3 (5)	N2—C205—H20R	109.00
O2'—C13'—C12'	122.2 (5)	N2—C205—H20S	109.00
C12'—C13'—C14'	115.6 (4)	C206—C205—H20R	109.00
S3'—C14'—C13'	119.5 (3)	C206—C205—H20S	109.00
S3'—C14'—C15'	119.6 (4)	H20R—C205—H20S	108.00
C13'—C14'—C15'	120.9 (4)	C205—C206—H20T	110.00
C14'—C15'—C16'	123.7 (5)	C205—C206—H20U	110.00
C11'—C16'—C15'	115.6 (5)	C205—C206—H20V	109.00
C15'—C16'—C17'	122.3 (6)	H20T—C206—H20U	109.00
C11'—C16'—C17'	121.9 (6)	H20T—C206—H20V	109.00
C16'—C17'—C19'	108.3 (6)	H20U—C206—H20V	109.00
C18'—C17'—C20'	106.0 (7)	N2—C207—H20Y	109.00
C16'—C17'—C20'	108.9 (6)	N2—C207—H20Z	108.00
C18'—C17'—C19'	108.3 (10)	C208—C207—H20Y	108.00
C16'—C17'—C18'	112.4 (6)	C208—C207—H20Z	108.00
C19'—C17'—C20'	113.0 (10)	H20Y—C207—H20Z	107.00
C22'—C21'—C26'	122.6 (5)	C207—C208—H2QW	109.00
S3'—C22'—C23'	120.8 (4)	C207—C208—H20Q	109.00
S3'—C22'—C21'	118.4 (4)	C207—C208—H20W	109.00
C21'—C22'—C23'	120.7 (5)	H2QW—C208—H20Q	109.00
O3'—C23'—C22'	123.9 (5)	H2QW—C208—H20W	110.00
O3'—C23'—C24'	118.7 (5)	H20Q—C208—H20W	109.00
			
C2—S1—C34—C33	−132.0 (5)	C42—C43—C48—C47	179.9 (6)
C2—S1—C34—C35	42.1 (5)	C42—C43—C44—C45	−179.5 (6)
C34—S1—C2—C3	59.3 (5)	C48—C43—C44—C45	0.7 (9)
C34—S1—C2—C1	−119.7 (5)	C43—C44—C45—C46	−1.1 (11)
C4—S2—C12—C11	−90.9 (5)	C44—C45—C46—C47	1.1 (12)
C4—S2—C12—C13	95.8 (4)	C45—C46—C47—C48	−0.6 (12)
C12—S2—C4—C3	−70.4 (4)	C46—C47—C48—C43	0.3 (11)
C12—S2—C4—C5	116.5 (4)	C2'—C1'—C6'—C7'	178.7 (6)
C22—S3—C14—C15	89.6 (4)	C6'—C1'—C2'—C3'	−1.3 (9)
C14—S3—C22—C21	−106.3 (5)	C2'—C1'—C6'—C5'	−0.3 (9)
C22—S3—C14—C13	−93.6 (4)	C6'—C1'—C2'—S1'	177.4 (5)
C14—S3—C22—C23	76.4 (4)	C1'—C2'—C3'—O1'	−177.0 (5)
C32—S4—C24—C25	109.1 (4)	S1'—C2'—C3'—C4'	−176.2 (4)
C32—S4—C24—C23	−67.1 (4)	C1'—C2'—C3'—C4'	2.5 (8)
C24—S4—C32—C31	−39.0 (4)	S1'—C2'—C3'—O1'	4.3 (7)
C24—S4—C32—C33	135.1 (4)	C2'—C3'—C4'—C5'	−2.3 (8)
C34'—S1'—C2'—C1'	−118.2 (5)	O1'—C3'—C4'—C5'	177.2 (5)
C34'—S1'—C2'—C3'	60.5 (5)	C2'—C3'—C4'—S2'	−176.5 (4)
C2'—S1'—C34'—C35'	41.9 (5)	O1'—C3'—C4'—S2'	2.9 (8)
C2'—S1'—C34'—C33'	−132.0 (5)	C3'—C4'—C5'—C6'	0.8 (9)
C12'—S2'—C4'—C3'	−70.9 (5)	S2'—C4'—C5'—C6'	175.2 (5)
C4'—S2'—C12'—C11'	−90.7 (5)	C4'—C5'—C6'—C7'	−178.5 (6)
C4'—S2'—C12'—C13'	96.3 (5)	C4'—C5'—C6'—C1'	0.5 (9)
C12'—S2'—C4'—C5'	114.7 (5)	C1'—C6'—C7'—C10'	133.5 (7)
C14'—S3'—C22'—C21'	−106.6 (4)	C5'—C6'—C7'—C8'	−166.0 (7)
C14'—S3'—C22'—C23'	77.0 (4)	C5'—C6'—C7'—C9'	69.7 (8)
C22'—S3'—C14'—C15'	90.0 (5)	C1'—C6'—C7'—C9'	−109.3 (7)
C22'—S3'—C14'—C13'	−93.6 (4)	C5'—C6'—C7'—C10'	−47.5 (9)
C32'—S4'—C24'—C25'	109.3 (4)	C1'—C6'—C7'—C8'	15.1 (10)
C24'—S4'—C32'—C31'	−39.8 (4)	C16'—C11'—C12'—S2'	−171.1 (5)
C32'—S4'—C24'—C23'	−66.6 (4)	C12'—C11'—C16'—C15'	5.1 (8)
C24'—S4'—C32'—C33'	134.6 (4)	C12'—C11'—C16'—C17'	−178.4 (6)
C41—O4—C33—C34	91.7 (5)	C16'—C11'—C12'—C13'	1.8 (9)
C41—O4—C33—C32	−88.4 (5)	C11'—C12'—C13'—O2'	170.8 (5)
C33—O4—C41—C42	177.2 (5)	C11'—C12'—C13'—C14'	−8.3 (7)
C6—C1—C2—C3	−1.1 (9)	S2'—C12'—C13'—O2'	−16.4 (6)
C2—C1—C6—C5	−1.2 (9)	S2'—C12'—C13'—C14'	164.5 (4)
C2—C1—C6—C7	179.3 (6)	O2'—C13'—C14'—S3'	12.7 (7)
C6—C1—C2—S1	177.8 (5)	O2'—C13'—C14'—C15'	−171.0 (5)
C1—C2—C3—C4	3.5 (8)	C12'—C13'—C14'—C15'	8.1 (7)
S1—C2—C3—C4	−175.5 (4)	C12'—C13'—C14'—S3'	−168.2 (4)
C1—C2—C3—O1	−176.5 (5)	S3'—C14'—C15'—C16'	174.9 (5)
S1—C2—C3—O1	4.6 (7)	C13'—C14'—C15'—C16'	−1.5 (9)
C2—C3—C4—S2	−176.4 (4)	C14'—C15'—C16'—C17'	178.2 (6)
C2—C3—C4—C5	−3.5 (7)	C14'—C15'—C16'—C11'	−5.3 (9)
O1—C3—C4—S2	3.6 (7)	C15'—C16'—C17'—C20'	−54.5 (9)
O1—C3—C4—C5	176.5 (5)	C15'—C16'—C17'—C18'	−171.7 (8)
C3—C4—C5—C6	1.3 (8)	C11'—C16'—C17'—C19'	−107.6 (10)
S2—C4—C5—C6	174.3 (5)	C15'—C16'—C17'—C19'	68.7 (11)
C41'—O4'—C33'—C34'	93.0 (5)	C11'—C16'—C17'—C18'	12.0 (10)
C41'—O4'—C33'—C32'	−87.6 (5)	C11'—C16'—C17'—C20'	129.2 (8)
C33'—O4'—C41'—C42'	176.7 (4)	C22'—C21'—C26'—C27'	−177.3 (5)
C4—C5—C6—C7	−179.4 (5)	C26'—C21'—C22'—S3'	−178.3 (5)
C4—C5—C6—C1	1.2 (9)	C22'—C21'—C26'—C25'	1.7 (9)
C1—C6—C7—C8	5.1 (10)	C26'—C21'—C22'—C23'	−1.8 (9)
C5—C6—C7—C8	−174.4 (7)	S3'—C22'—C23'—O3'	−2.2 (7)
C1—C6—C7—C10	125.9 (8)	C21'—C22'—C23'—C24'	1.7 (7)
C5—C6—C7—C10	−53.5 (9)	S3'—C22'—C23'—C24'	178.1 (4)
C1—C6—C7—C9	−116.3 (7)	C21'—C22'—C23'—O3'	−178.6 (5)
C5—C6—C7—C9	64.3 (8)	O3'—C23'—C24'—S4'	−5.6 (6)
C16—C11—C12—C13	2.0 (8)	C22'—C23'—C24'—C25'	−1.8 (7)
C16—C11—C12—S2	−171.2 (4)	O3'—C23'—C24'—C25'	178.6 (4)
C12—C11—C16—C15	5.1 (8)	C22'—C23'—C24'—S4'	174.1 (4)
C12—C11—C16—C17	−177.6 (5)	C23'—C24'—C25'—C26'	1.8 (8)
S2—C12—C13—C14	164.4 (4)	S4'—C24'—C25'—C26'	−174.0 (4)
C11—C12—C13—C14	−8.8 (7)	C24'—C25'—C26'—C27'	177.2 (5)
S2—C12—C13—O2	−15.9 (7)	C24'—C25'—C26'—C21'	−1.7 (8)
C11—C12—C13—O2	171.0 (5)	C21'—C26'—C27'—C29'	−61.8 (8)
O2—C13—C14—S3	12.2 (6)	C25'—C26'—C27'—C30'	−1.7 (8)
O2—C13—C14—C15	−171.0 (4)	C25'—C26'—C27'—C28'	−120.6 (6)
C12—C13—C14—C15	8.7 (6)	C21'—C26'—C27'—C28'	58.3 (8)
C12—C13—C14—S3	−168.1 (4)	C25'—C26'—C27'—C29'	119.3 (6)
C13—C14—C15—C16	−1.9 (7)	C21'—C26'—C27'—C30'	177.2 (6)
S3—C14—C15—C16	174.9 (4)	C36'—C31'—C32'—S4'	174.1 (4)
C14—C15—C16—C17	177.5 (5)	C32'—C31'—C36'—C37'	−177.8 (5)
C14—C15—C16—C11	−5.2 (7)	C36'—C31'—C32'—C33'	−0.2 (7)
C11—C16—C17—C18	17.2 (9)	C32'—C31'—C36'—C35'	−0.2 (7)
C11—C16—C17—C19	−104.6 (7)	S4'—C32'—C33'—C34'	−173.0 (4)
C15—C16—C17—C18	−165.6 (7)	S4'—C32'—C33'—O4'	7.6 (6)
C11—C16—C17—C20	136.3 (6)	C31'—C32'—C33'—O4'	−178.2 (4)
C15—C16—C17—C19	72.5 (7)	C31'—C32'—C33'—C34'	1.3 (7)
C15—C16—C17—C20	−46.6 (8)	O4'—C33'—C34'—S1'	−8.5 (6)
C26—C21—C22—S3	−179.2 (5)	C32'—C33'—C34'—C35'	−1.9 (7)
C26—C21—C22—C23	−2.0 (9)	C32'—C33'—C34'—S1'	172.1 (4)
C22—C21—C26—C25	2.5 (9)	O4'—C33'—C34'—C35'	177.6 (5)
C22—C21—C26—C27	−177.5 (6)	C33'—C34'—C35'—C36'	1.4 (8)
C21—C22—C23—C24	1.4 (7)	S1'—C34'—C35'—C36'	−172.4 (4)
S3—C22—C23—C24	178.6 (4)	C34'—C35'—C36'—C31'	−0.4 (8)
C21—C22—C23—O3	−178.5 (5)	C34'—C35'—C36'—C37'	177.2 (5)
S3—C22—C23—O3	−1.3 (7)	C31'—C36'—C37'—C39'	−104.3 (8)
C22—C23—C24—C25	−1.6 (7)	C31'—C36'—C37'—C40'	134.9 (6)
O3—C23—C24—C25	178.3 (5)	C31'—C36'—C37'—C38'	16.0 (9)
C22—C23—C24—S4	174.5 (4)	C35'—C36'—C37'—C39'	78.2 (9)
O3—C23—C24—S4	−5.6 (6)	C35'—C36'—C37'—C40'	−42.6 (8)
C23—C24—C25—C26	2.3 (9)	C35'—C36'—C37'—C38'	−161.6 (7)
S4—C24—C25—C26	−173.8 (5)	O4'—C41'—C42'—O5'	0.4 (8)
C24—C25—C26—C27	177.3 (6)	O4'—C41'—C42'—C43'	−177.3 (5)
C24—C25—C26—C21	−2.7 (9)	O5'—C42'—C43'—C44'	−0.4 (9)
C21—C26—C27—C30	179.6 (8)	O5'—C42'—C43'—C48'	−179.7 (7)
C25—C26—C27—C29	118.4 (8)	C41'—C42'—C43'—C44'	177.3 (5)
C21—C26—C27—C29	−61.6 (8)	C41'—C42'—C43'—C48'	−2.0 (9)
C21—C26—C27—C28	58.9 (8)	C42'—C43'—C44'—C45'	−179.8 (6)
C25—C26—C27—C28	−121.1 (7)	C48'—C43'—C44'—C45'	−0.6 (9)
C25—C26—C27—C30	−0.4 (10)	C42'—C43'—C48'—C47'	−179.7 (6)
C32—C31—C36—C35	−0.1 (7)	C44'—C43'—C48'—C47'	1.1 (9)
C32—C31—C36—C37	−179.2 (4)	C43'—C44'—C45'—C46'	0.6 (10)
C36—C31—C32—C33	−0.1 (7)	C44'—C45'—C46'—C47'	−1.1 (11)
C36—C31—C32—S4	174.0 (3)	C45'—C46'—C47'—C48'	1.6 (11)
C31—C32—C33—C34	1.1 (7)	C46'—C47'—C48'—C43'	−1.6 (9)
C31—C32—C33—O4	−178.8 (4)	C103—N1—C101—C102	−59.0 (13)
S4—C32—C33—O4	7.2 (6)	C105—N1—C101—C102	171.7 (12)
S4—C32—C33—C34	−173.0 (4)	C107—N1—C101—C102	59.9 (13)
C32—C33—C34—C35	−1.9 (7)	C101—N1—C103—C104	−51.6 (13)
O4—C33—C34—S1	−8.0 (6)	C105—N1—C103—C104	70.0 (14)
C32—C33—C34—S1	172.2 (4)	C107—N1—C103—C104	−170.0 (11)
O4—C33—C34—C35	178.0 (5)	C101—N1—C105—C106	179.2 (13)
C33—C34—C35—C36	1.7 (8)	C103—N1—C105—C106	55.6 (17)
S1—C34—C35—C36	−172.4 (4)	C107—N1—C105—C106	−66.6 (16)
C34—C35—C36—C31	−0.7 (7)	C101—N1—C107—C108	71.3 (15)
C34—C35—C36—C37	178.4 (5)	C103—N1—C107—C108	−169.4 (14)
C35—C36—C37—C40	−41.7 (7)	C105—N1—C107—C108	−41.0 (17)
C31—C36—C37—C40	137.3 (6)	C203—N2—C201—C202	−60.6 (12)
C31—C36—C37—C38	17.0 (8)	C205—N2—C201—C202	178.7 (10)
C31—C36—C37—C39	−102.9 (7)	C207—N2—C201—C202	59.9 (12)
C35—C36—C37—C38	−162.1 (6)	C201—N2—C203—C204	−53.0 (11)
C35—C36—C37—C39	78.0 (7)	C205—N2—C203—C204	63.3 (11)
O4—C41—C42—O5	3.1 (8)	C207—N2—C203—C204	−173.9 (10)
O4—C41—C42—C43	−176.5 (5)	C201—N2—C205—C206	−175.4 (9)
C41—C42—C43—C44	177.2 (5)	C203—N2—C205—C206	64.0 (11)
C41—C42—C43—C48	−3.0 (9)	C207—N2—C205—C206	−57.6 (12)
O5—C42—C43—C48	177.5 (7)	C201—N2—C207—C208	67.7 (14)
O5—C42—C43—C44	−2.3 (10)	C203—N2—C207—C208	−170.1 (12)
C44—C43—C48—C47	−0.3 (9)	C205—N2—C207—C208	−46.8 (15)

### Hydrogen-bond geometry (Å, º)

**Table:**

*D*—H···*A*	*D*—H	H···*A*	*D*···*A*	*D*—H···*A*
O1—H1*O*···S2	0.82	2.61	3.110 (4)	121
O1—H1*O*···O2	0.82	1.85	2.581 (6)	147
O2—H2*O*···Cl2	0.82	2.30	3.048 (10)	152
O2—H2*O*···S2	0.82	2.70	3.051 (4)	108
O3—H3*O*···S3	0.82	2.57	3.077 (4)	121
O3—H3*O*···O2	0.82	1.91	2.567 (6)	136
O1′—H6*O*···S2′	0.82	2.60	3.105 (4)	121
O1′—H6*O*···O2′	0.82	1.85	2.580 (5)	147
O1′—H6*O*···Cl1^i^	0.82	2.81	3.338 (11)	124
O2′—H7*O*···S2′	0.82	2.68	3.044 (4)	109
O2′—H7*O*···Cl1^i^	0.82	2.27	3.006 (10)	150
O3′—H8*O*···S3′	0.82	2.57	3.078 (4)	121
O3′—H8*O*···O2′	0.82	1.90	2.567 (5)	138
C103—H10*L*···Cl2	0.97	2.43	3.397 (17)	174
C106—H10*V*···Cl2	0.96	2.64	3.382 (19)	135
C202—H20*I*···O5	0.96	2.58	3.459 (16)	152
C203—H20*L*···Cl1^ii^	0.97	2.41	3.373 (15)	173
C204—H20*P*···Cl2^ii^	0.96	2.78	3.63 (2)	147
C206—H20*V*···Cl1^ii^	0.96	2.55	3.353 (18)	141
C41—H41*A*···O3	0.97	2.45	3.318 (6)	149
C41—H41*B*···O1	0.97	2.46	3.253 (6)	139
C41′—H41*C*···S4′	0.97	2.87	3.414 (6)	116
C41′—H41*C*···O3′	0.97	2.45	3.313 (6)	148
C41′—H41*D*···O1′	0.97	2.47	3.265 (6)	139
C44—H44···O2^iii^	0.93	2.54	3.431 (7)	160
C44′—H44′···O2′^iv^	0.93	2.57	3.447 (6)	158
C47—H47···O4^v^	0.93	2.55	3.368 (6)	146
C47—H47···O5^v^	0.93	2.45	3.255 (7)	145
C47′—H47′···O4′^vi^	0.93	2.57	3.380 (6)	146
C47′—H47′···O5′^vi^	0.93	2.47	3.277 (7)	145

Symmetry codes: (i) −*x*+1, *y*+1/2, −*z*+1; (ii) *x*, *y*+1, *z*; (iii) −*x*+2, *y*+1/2, −*z*+2; (iv) −*x*+1, *y*−1/2, −*z*; (v) −*x*+2, *y*−1/2, −*z*+2; (vi) −*x*+1, *y*+1/2, −*z*.
