# Supplementary figures and images for: Crystal structure of the inclusion complex 25-benzo­ylmeth­oxy-5,11,17,23-tetra-tert-butyl-26,27,28-trihy­droxy-2,8,14,20-tetra­thia­calix[4]arene–tetra­ethyl­ammonium chloride (1/1)
Source: Acta Crystallogr E Crystallogr Commun. 2015 Oct 10;71(Pt 11):o830–1. doi: 10.1107/S2056989015018617 (PMC4645050; doi:10.1107/S2056989015018617)

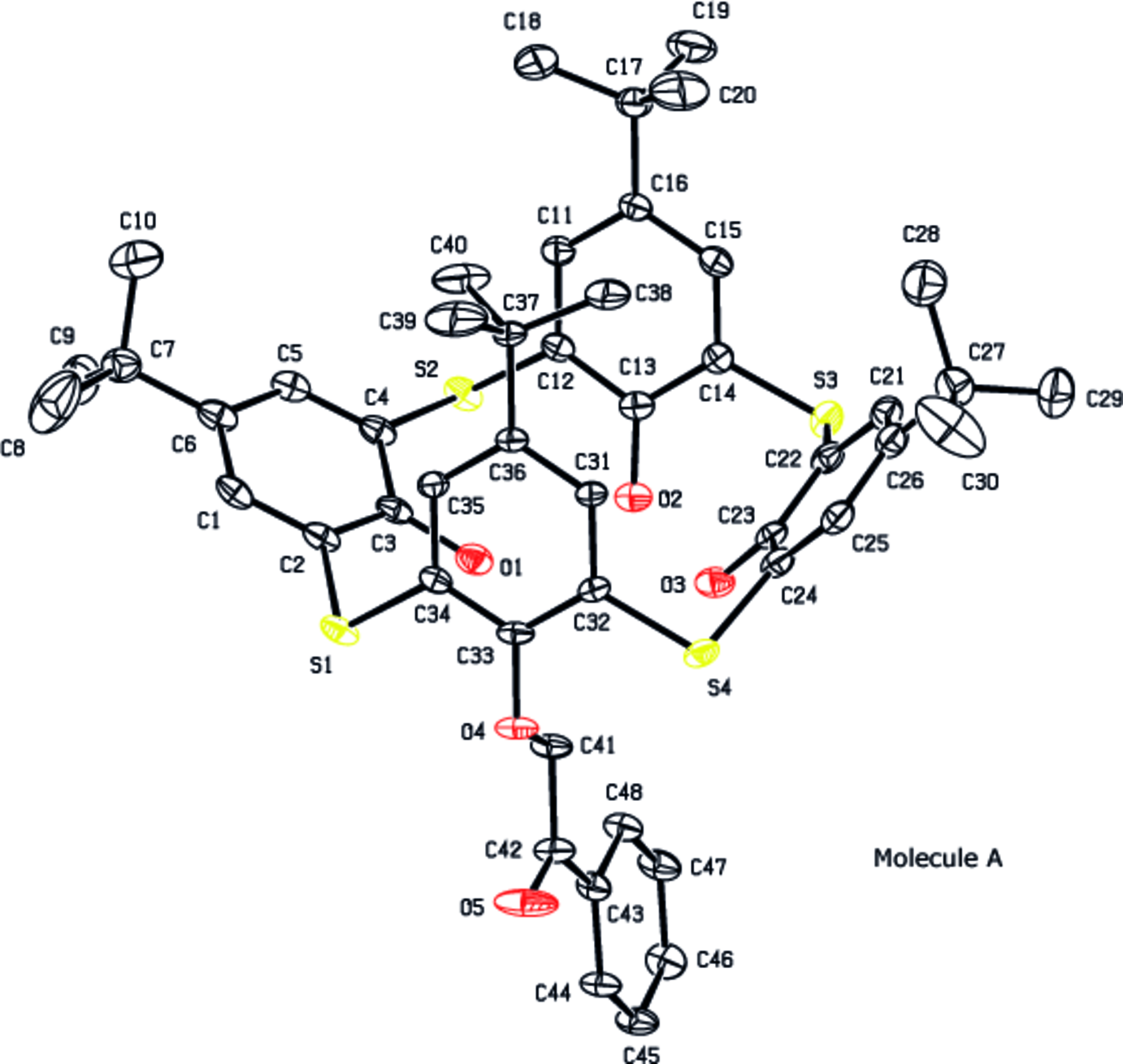

Supplement: Supplementary file 3 [file e-71-0o830-fig1.tif]

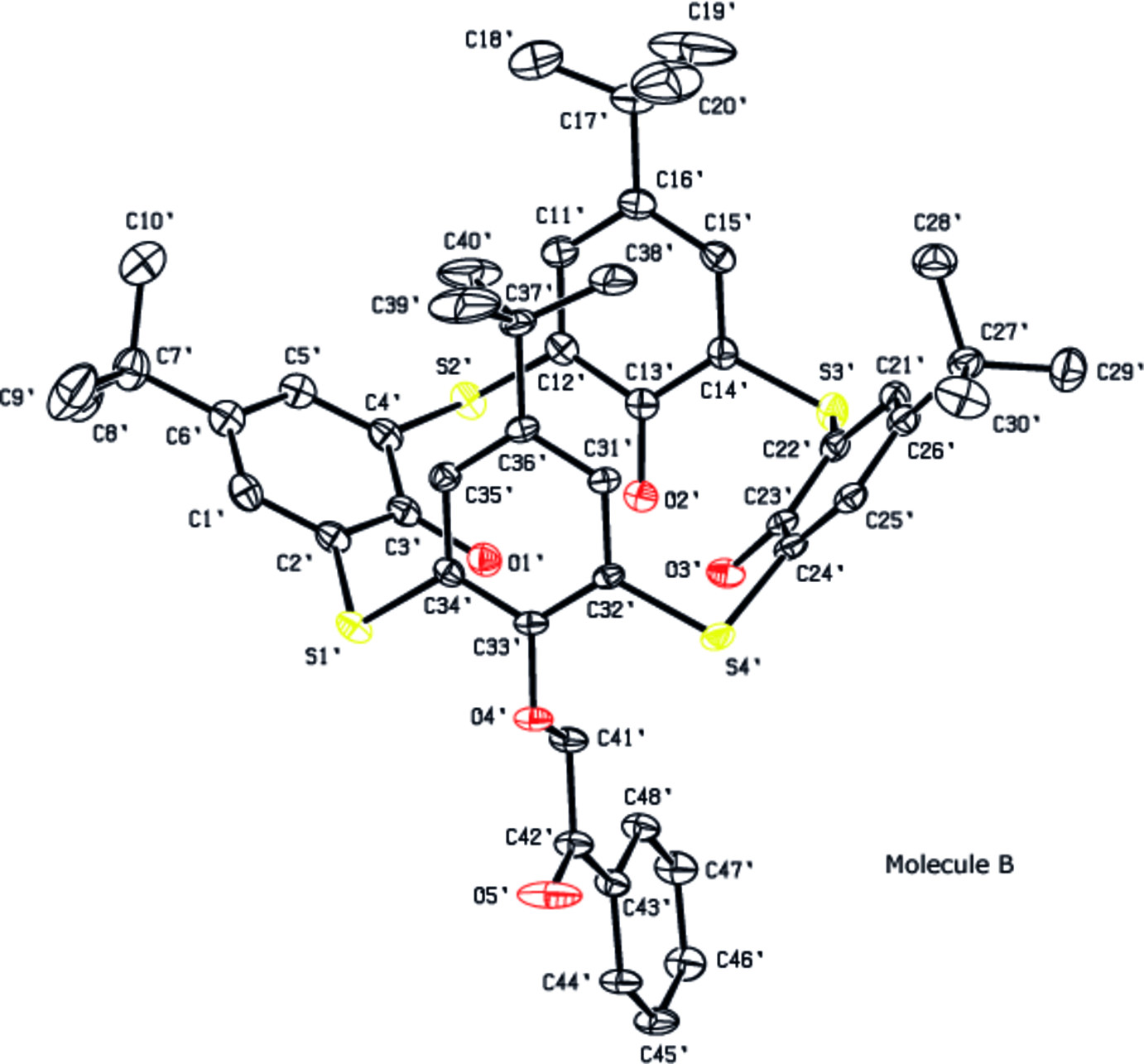

Supplement: Supplementary file 4 [file e-71-0o830-fig2.tif]

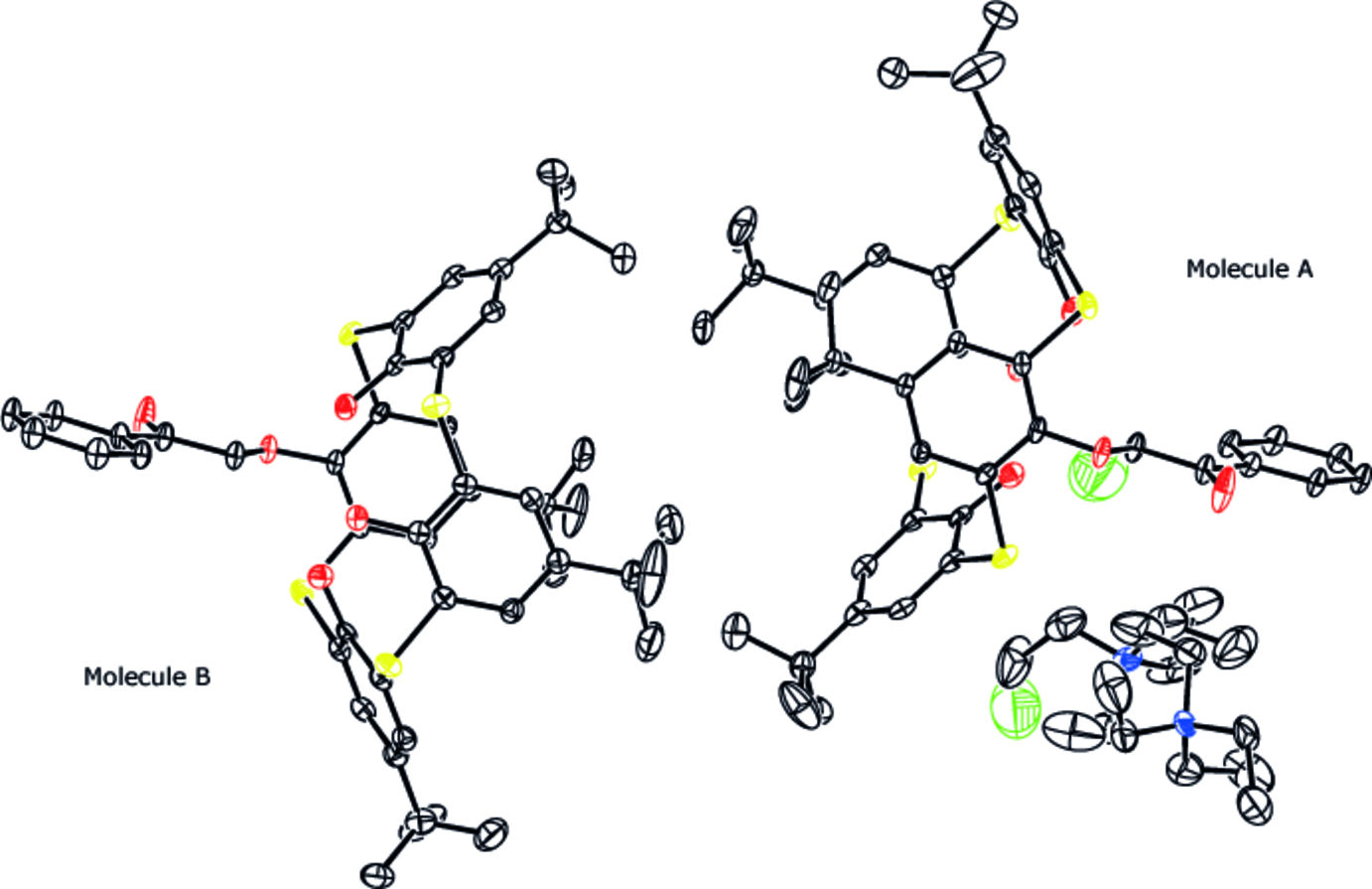

Supplement: Supplementary file 5 [file e-71-0o830-fig3.tif]

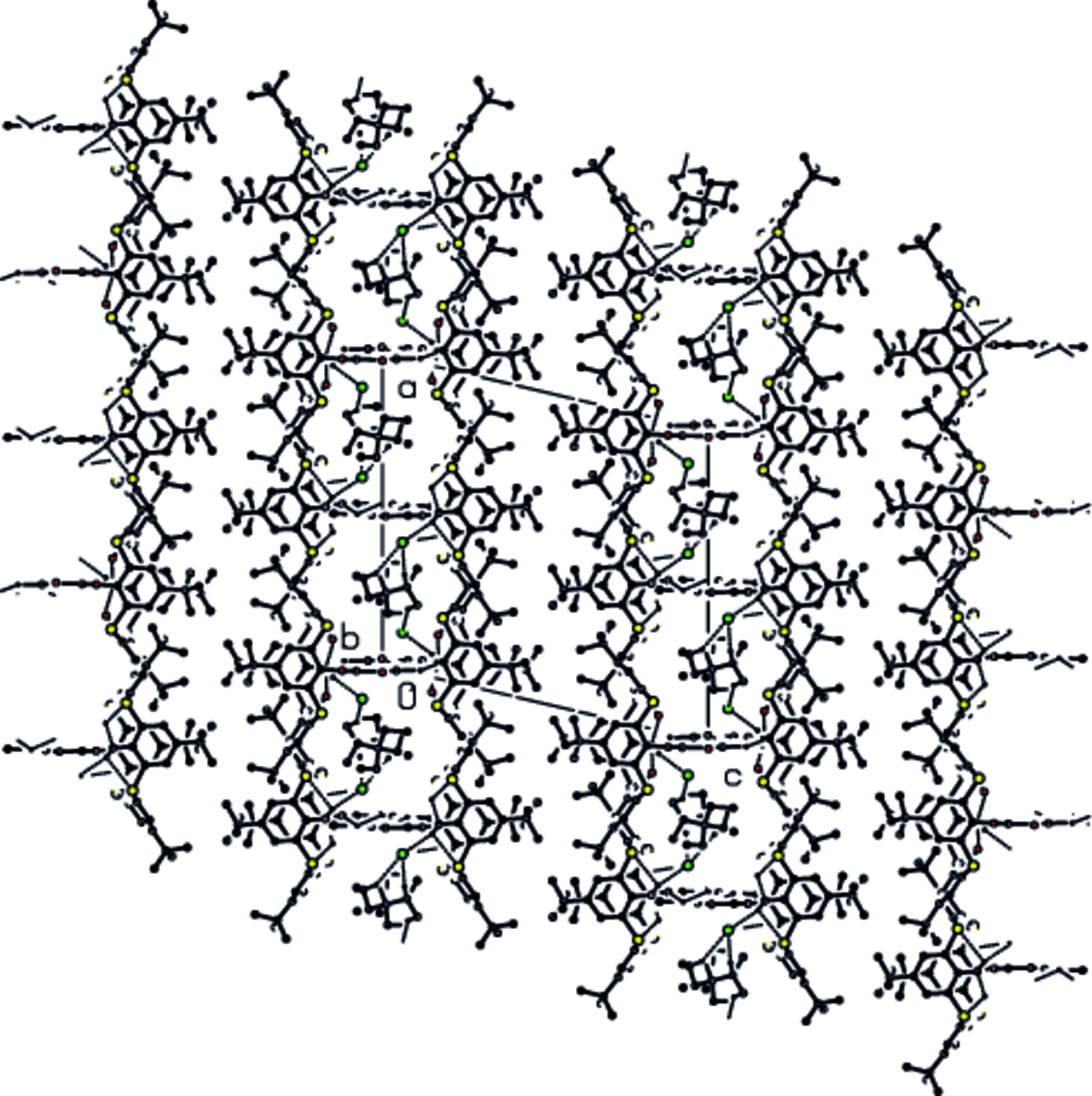

Supplement: Supplementary file 6 [file e-71-0o830-fig4.tif]
